# MSDDG: Multi-scale dual-discriminator GAN for point cloud completion of plant

**DOI:** 10.1016/j.plaphe.2026.100218

**Published:** 2026-04-23

**Authors:** Qingguang Chen, Jiajin Liu, Hongwei Sun, Ting Sun, Wenyu Zhang, Hang Lu, Yingying Pan, Wenhan Luo, Lianjie Chen, Jie Ying, Simin Kang, Jingcheng Zhang, Kaihua Wu

**Affiliations:** a School of Automation, Hangzhou Dianzi University, Hangzhou, 310018, China; b Jiangsu Academy of Agricultural Sciences Wuxi Branch, Shicheng Road 388, Wuxi, 214174, China

**Keywords:** Point cloud completion, Multi-view silhouette, Multi-scale, GAN, Dual-discriminator

## Abstract

Plant 3D reconstruction using optical imaging often suffers from incomplete point clouds due to viewpoint occlusion and sensor limitations. This incompleteness hinders accurate structural representation and subsequent feature extraction for plant analysis. To address these challenges, we propose a Multi-Scale Dual-Discriminator Generative Adversarial Network (MSDDG) for plant point cloud completion. A multi-scale point cloud generator (MSPG) that integrates local and global features from raw incomplete point clouds is used for MSDDG to reconstruct complete shapes. The dual-discriminators—a multi-view projected silhouette discriminator and a spatial distance discriminator—are designed to ensure geometric realism and spatial plausibility from multiple perspectives. To train MSDDG, we created the Plant4L dataset containing four plant species (sunflower, pumpkin, luffa, and eggplant) with high-quality 3D models augmented via 3D thin plate spline transformations and virtual occlusion simulation to generate incomplete point clouds and multi-view silhouettes. Experimental results on Plant4L demonstrate that MSDDG achieves superior completion performance, with Chamfer Distance (CD), Hausdorff Distance (HD), and Uniformity Chamfer Distance (UCD) all below 0.41. Comparative evaluations confirm MSDDG's superiority over previous point cloud completion methods. The application of MSDDG for 3D reconstruction from single view further validate its effectiveness in restoring occluded plant structures.

## 1 Introduction

3D reconstruction of plants is the key data of individual crop analysis research. The visualization and quantitative analysis of the spatial structure of plants through a point cloud can reflect the 3D expression of plant genes in time series and its regional differentiation characteristics and evolution rules [1]. In recent years, researchers have widely utilized optical imaging technology to obtain point cloud data, and combined with three-dimensional reconstruction techniques such as multi-view structure (MVS) [[2], [3], [4]], structure from motion (SFM) [5], point cloud registration and fusion [6,7], to construct three-dimensional models of plants and apply them to various aspects of smart agriculture, such as extraction of plant phenotypic parameters, crop growth monitoring, and precise planting management. However, due to viewpoint occlusion and sensor limitations, the point cloud data obtained through optical imaging in the three-dimensional reconstruction process often suffer from missing data [8], which leads to the absence of information about the internal structure of the plant [9]. The three-dimensional spatial structure of plants is complex. This complexity poses challenges to accurately and completely describing individual plant structures [10].

To address the issue of incomplete point clouds, point cloud completion technology predicts and reconstructs the complete geometric structure from the partially missing point clouds, thereby enhancing the integrity and practicality of the three-dimensional model. Previous researchers have proposed various methods for completing the missing three-dimensional point cloud data, including voxel-based point cloud completion, deep learning-based point cloud completion, and projection shape constraints point cloud completion.

The voxel-based point cloud completion algorithm refers to the region-growing process in the voxel space that can propagate the structural prior from the observed area to the occluded area, ensuring topological consistency throughout the completion process [11]. This method possesses four ideal attributes: range uncertainty representation, independent update of increments and sequence, certain temporal and spatial efficiency, and unrestricted topological types [12]. Choudhury et al. utilized voxelized grids based on multi-view images to calculate voxel overlap rates and perform clustering for plant segmentation and detection, demonstrating the semantic consistency of voxel representation in plant structures [13]. Yang Lin proposed a method combining voxelization and region growing to reconstruct oilseed plant point clouds, leveraging voxel geometry and color cues to guide growth direction [14].

However, voxel-based methods typically suffer from high computational cost and memory consumption when pursuing high-resolution reconstructions. They also struggle with representing fine structural details, especially in sparse or occluded regions. These limitations make it difficult to balance topological continuity with detailed realism, reducing their scalability in practical plant phenotyping tasks.

The point cloud completion method based on deep learning integrates the idea of deep learning with the analysis of point clouds. Qi proposed PointNet [15] and PointNet++ [16], which use encoder-decoder structures to process unordered point sets and extract hierarchical features. Methods like PCN [17], VRCNet [18], and ShapInversion [19] convert incomplete inputs into feature vectors to generate plausible completions. GAN-based models like RL-GAN-NET [20] and FoldingNet [21] offer additional realism through adversarial learning or folding-based decoding. Other enhancements include attention-based SA-Net [22], geometry-aware PF-Net [23], and Fu-Net with feature fusion [24]. Li et al. proposed a leaf-level completion network [25], while Zeng's work introduced multi-scale geometric encoding via Transformer [26]. Chen's method improved phenotype prediction using single-leaf completion techniques [27].

Despite these advances, most deep learning methods tend to lose either local geometric precision or global structural consistency, particularly when the missing regions are large or complex. The predicted shapes often deviate from the ground-truth distribution, especially in dense foliage or irregular topologies. Moreover, the learned priors are limited by dataset diversity, making it challenging to generalize to novel plant morphologies.

The point cloud completion method based on projection shape constraints combines point clouds and 2D images to form multi-modal inputs. By leveraging 2D silhouette projections, the model can better reason about occluded structures based on global geometric context [28]. Yang et al. designed a GAN-based model for occluded tomato fruit recovery [29], while Lou used bird's-eye RGB views to guide lettuce leaf completion [30]. Li proposed Cascade Leaf Segmentation and Completion Network(CLSCN) and Fragmental Leaf Point–cloud Reconstruction Algorithm(FLPRA) to complete point clouds using leaf mask predictions as projection constraints [31].

These approaches effectively constrain 3D predictions within the bounds of observed image regions, but they heavily rely on precise image–point cloud alignment and are sensitive to view selection and segmentation quality. Furthermore, they typically overlook how to incorporate multi-scale geometry or adversarial realism into the reconstruction pipeline.

Therefore, to address these limitations, we propose a novel point cloud completion framework, MSDDG, which combines multi-view silhouette guidance with multi-scale geometry-aware generative learning. Our method introduces projection priors while maintaining structural realism via a dual-discriminator adversarial design.

This study proposes a generative point cloud completion network with multi-view silhouette constraint-MSDDG(Multi-scale dual-discriminator GAN), for the completion of three-dimensional point clouds of plants. To train the network, complete point cloud data of the plants were collected using existing methods and devices. The geometric shape of the plants was expanded through non-rigid transformation. A point cloud completion dataset covering different morphologies of the same type of plants was constructed, and a virtual camera was used to divide the visible and occluded areas and generate corresponding multi-view projection silhouette images. For the occluded areas, MSDDG uses a multi-resolution generative adversarial network to predict the missing parts, and uses the projection silhouette images generated from the density estimation of the complete point cloud as prior constraints to guide the network to generate a structurally reasonable completed point cloud model. The multi-scale GAN with dual discriminator point cloud completion method proposed in this paper can effectively restore the fine structures of different organs of plants. This study mainly includes the following contributions:

• To address the lack of high-quality plant datasets for point cloud completion, we construct a novel 3D dataset named **Plant4L**, which includes four types of plant species. By applying non-rigid transformations to simulate natural morphological variation, this dataset provides rich shape diversity and serves as a solid benchmark for evaluating completion performance under realistic conditions.
• To improve the generation of complete plant structures from incomplete data, we propose a **multi-scale point cloud generator (MSPG).** It integrates both global and local features extracted by a multi-resolution encoder (MRE), enabling the network to preserve fine details while maintaining overall geometric consistency.
• To ensure that the generated point clouds are both geometrically plausible and visually realistic from multiple views, we design a **dual-discriminator framework.** It combines a multi-view projection silhouette discriminator and a spatial distance discriminator. This adversarial structure leverages 2D projection priors and 3D spatial metrics to constrain the generation process and improve fidelity.
• Extensive experiments on the Plant4L dataset and real application scenarios demonstrate that our proposed **MSDDG framework significantly outperforms** existing methods in terms of completeness, accuracy, and robustness, especially in complex occlusion scenarios common in plant environments.

## 2 Method

To address the issue of dataset deficiency in the public datasets for plant point cloud completion, we used a self-constructed multi-view 3D reconstruction experimental device to reconstruct 4 different species of 3D initial point cloud models of multiple plants. We also constructed a dataset named Plant4L for plant 3D point cloud completion through data augmentation techniques. Using a virtual imaging system, we formed corresponding data such as missing point clouds, visible point clouds, and point cloud projection contour images under different views. We designed a generative adversarial network structure containing multi-scale generators and dual-discriminators. By training the network using the Plant4L dataset, we obtained a generator with strong perception of plant geometry and spatial distribution for point cloud completion.

### 2.1 Dataset construction

In order to obtain the initial complete three-dimensional reconstruction results of different species of plant objects, our reported 3D reconstruction platform is utilized [32]. After acquiring the calibration of the sensor, depth images of the measured plants are collected from multiple views, and the point cloud is extracted using the point cloud denoising method based on multi-view projection contour consistency constraints [33]. Then, the complete three-dimensional point cloud model of the plant is reconstructed through multi-perspective registration and fusion.

#### 2.1.1 Data acquisition

The three-dimensional point cloud of the plant represents the spatial distribution of the three-dimensional structure of the plant. Although its different forms indicate the randomness of growth, from a global perspective, the entire structure still has significant morphological characteristics [34]: for example, in the case of sunflower seedlings, the first pair of leaves are opposite and the leaf surface has hairs, while subsequent leaves become alternate and have shapes of heart-shaped or oval-shaped. The stem is a straight cylindrical shape and the internodes are initially short [35].

The initial 3D data of the plants was collected using the RGB-D sensor of Intel RealSense L515 and the high-precision rotating platform TMC [36]. As shown in Fig. S1a, the device was fixed on a support, with an area of approximately $1.0\times 0.5\;m^{2}$, capable of covering the entire canopy and stem structure of the plants. The fixed height of the L515 could be adjusted according to the size of the plants, ensuring both completeness and accuracy. As shown in Fig. S1bc, fixed-resolution RGB and depth images were collected during data acquisition. a Based on the collected RGB images, the plant-area mask shown in Fig. S1d was annotated using an annotation tool to remove the soil and the portions below the soil surface. The 3D reconstruction system device is shown in Fig. S2.

By using the depth maps collected after calibrating the sensors, point clouds from multiple views can be obtained. To address the issue that depth camera often generates a large amount of noise when generating point clouds, a point cloud filtering algorithm based on multi-view 2D image contour constraints was employed. This algorithm utilized the plant contour information from multiple views’ 2D images to constrain the boundary of the plant point cloud in 3D space. As a result, noise-free and outlier-free point clouds for each perspective were obtained, and multi-view point cloud registration was achieved based on the calibration results of the rotation axis to acquire the complete 3D point cloud of the plant. The multi-view point cloud registration formula is as follows:

(1) $$\left[\begin{array}{c} x_{i}^{w}\\ y_{i}^{w}\\ z_{i}^{w}\\ 1 \end{array}\right]=RT^{v}\cdot \left[\begin{array}{c} x_{i}^{v}\\ y_{i}^{v}\\ z_{i}^{v}\\ 1 \end{array}\right]$$

where, $RT^{v}$ represents the rotation and translation matrix that transforms the camera pose of the v th viewpoint to the camera pose of the first viewpoint; $\left(x_{i}^{w},y_{i}^{w},z_{i}^{w}\right)$ represents the coordinate of the i-th spatial point in the point cloud of the v th viewpoint in the camera coordinate system of the v th viewpoint; $\left(x_{i}^{v},y_{i}^{v},z_{i}^{v}\right)$ represents the coordinate of the i-th spatial point in the world coordinate system.

#### 2.1.2 Data augmentation

To further enrich the diversity of the three-dimensional reconstruction results of plants, we utilized the non-rigid transformation technique to perform deformation operations on specific leaves and stems, simulating the differences in the growth processes of plants in nature. The specific implementation process is shown in Fig. 1.

**Fig. 1:**
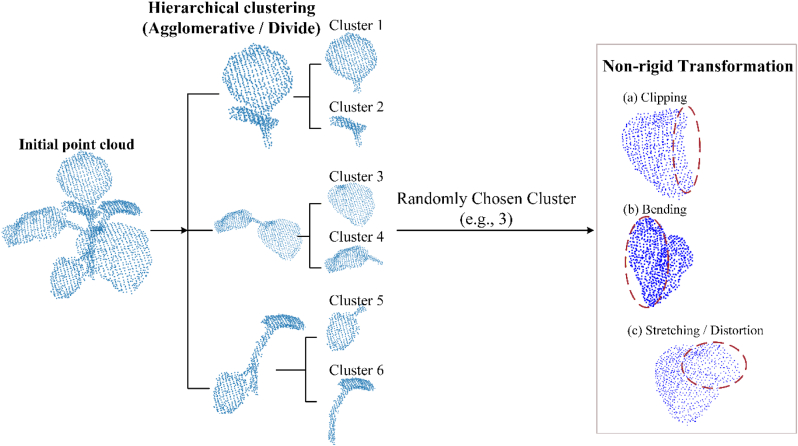
The process of non-rigid transformation of part of the point cloud after clustering, where different color indicates different cluster category. p denotes the initial point cloud while clustering. Hierarchical clustering can represent the final result as a tree structure. Point cloud (a)(b)(c) shows the schematic of three non-rigid transformation methods which based on clustered result. Each initial point cloud could generate a given number of new point clouds through a non-rigid transformation chosen by random clustering.

The augmentation of the sample quantity of the dataset Plant4L is achieved by diversifying the sample forms through non-rigid transformations of the 3D point cloud of the collected plants. The non-rigid transformation of the 3D point cloud of the plants refers to optimizing the global deformation while maintaining the rigidity of local regions, and ensuring that the rigid transformation error of each local neighborhood remains unchanged [37]. This step avoids the complexity of data collection and the large amount of work involved in 3D reconstruction, ensuring that the 3D point cloud data of the plants in the dataset have more diverse and distinct morphological features from the initial data.

For each plant point cloud, hierarchical clustering was performed to segment the data. Based on the clustering results, the point clouds corresponding to the stem and leaf regions of the plant were identified. For specific categories of point clouds, a non-rigid transformation method was applied to achieve local morphological adjustments. The region circled in red in Fig. 1 shows the point cloud obtained after applying non-rigid transformation to the initial data. This study employed hierarchical clustering to group point cloud data based on similarity. By constructing a hierarchical clustering structure, the stem and leaf regions of the plant point cloud were effectively distinguished. After determining an appropriate number of clusters, the core idea of hierarchical clustering is to iteratively merge similar points based on distance calculations, or to further divide an existing cluster into multiple sub-clusters. The final result can be represented as a tree structure. Hierarchical clustering mainly involves two components: distance measurement and a merging (or splitting) strategy during the clustering process. In this study, the distance was computed based on the average distance $d_{avg}$ between all point pairs within a cluster, using the following formula:

(2) $$d_{avg}\left(C_{i},C_{j}\right)=\frac{1}{\left|C_{i}\right|\left|C_{j}\right|}\sum_{p\in C_{i}}\sum_{p\in C_{j}}d\left(p,q\right)$$

where $C_{i},C_{j}$ denote the given clusters; $\left|C_{i}\right|,\left|C_{j}\right|$ represent the number of points in clusters $C_{i}$ and $C_{j}$, respectively; and d(p,q) indicates the distance between point p and point q. The clustering process begins by treating each point as an individual cluster. Then, the most similar clusters are gradually merged in an iterative manner. After each merging step, the distance between the newly formed cluster and the remaining clusters is defined as the distance between their respective centers. For each merged cluster $C_{i}\cup C_{j}$, the center $\phi_{ij}$ of the new cluster is calculated using the following formula:

(3) $$\phi_{ij}=\frac{\phi_{i}\left|C_{i}\right|+\phi_{j}\left|C_{j}\right|}{\left|C_{i}\right|+\left|C_{j}\right|}$$

where, $\phi_{i},\phi_{j}$ represent the centroids of cluster $C_{i}$ and cluster $C_{j}$ respectively.

Based on the point cloud clusters that have distinguished stems and leaves, a non-rigid transformation based on thin-plate spline function [38] is applied to the stems or leaves to obtain new morphological point cloud data. It can deform a set of source points(or shapes) into a set of target points, not only ensuring precise matching of transformation results at known points, but also generating transformations that are as smooth as possible in other areas. The non-rigid transformation of TPS can be expressed as:

(4) y=W·ψ(x)+Ax+b

where, y represents the transformed point cloud, x is the selected original point cloud, W is a weight matrix related to the positions of the control points, ψ(x) is a basis function indicating the distance between the input point and the control point, and A and b are parameters ensuring the rigid transformation of the point cloud. To model both radial and directional deformations of plant organs, the basis function is extended as a weighted combination of radial and tangential components. The radial component captures distance-related smooth deformations, while the tangential (or angular) component encodes direction-sensitive shape variations. Therefore, a weighted combination of Radial Basis Function (RBF) and tangential (angular) basis function is employed as the TPS basis. For any point x in the point cloud, the non-rigid TPS transformation with radial-tangential weighting can be expressed as:

(5) $$y=\sum_{i}W_{i}\left[\alpha \psi_{r}\left(x,x_{i}\right)+\beta \psi_{t}\left(x,x_{i}\right)\right]+Ax+b$$

where $x_{i}$ represents the i-th control point, and α and β are the weights for the radial basis function $\psi_{r}\left(x,x_{i}\right)$ and tangential (angular) basis function $\psi_{t}\left(x,x_{i}\right)$, respectively. These basis functions are computed as:

(6) $$\psi_{r}\left(x,x_{i}\right)={\left\|x-x_{i}\right\|}^{2}\;\log\left\|x-x_{i}\right\|$$

(7) $$\psi_{t}\left(x,x_{i}\right)=\frac{\left(x-x_{i}\right)}{\left\|x-x_{i}\right\|}\cdot n$$

where n denotes a unit direction vector defined for the tangential component, typically set as the point cloud normal computed via PCA. The above TPS non-rigid transformation with radial-tangential weighting can apply directionally aware morphological perturbations to key regions, such as leaves and stems, while maintaining local continuity and overall geometric consistency, thereby generating three-dimensional plant point clouds with diverse shapes and natural structures.

After clustering the complete point clouds of each plant and performing non-rigid transformations on the point clouds of the stem or leaf regions, a new point cloud with some differences in shape was roughly obtained. At the same time, plants with different structural configurations were combined. Using the open-source software Blender, the point clouds of the leaf and stem regions were manually selected. These selected point clouds were then bent, distorted, stretched, or clipped to achieve the desired transformations. Through non-rigid transformations of the point clouds, the expansion of different plant point cloud samples was realized to enhance the sample quantity of the dataset, and the dataset was divided into training set, test set and validation set.

### 2.2 Multi-scale dual-discriminator GAN for point cloud completion network

To address the challenge of incomplete plant point clouds caused by occlusions and sensor limitations[39], this study proposes a novel completion framework that incorporates multi-scale geometric understanding and multi-view projection priors. Existing completion methods often struggle to recover fine structural details or maintain contour consistency, especially in dense and complex plant architectures. To overcome these limitations, we design a Multi-scale Dual-Discriminator GAN (MSDDG) that leverages both 3D spatial features and 2D silhouette constraints to enhance the realism and completeness of the reconstructed point clouds.

The structure of the MSDDG network framework proposed in this study is shown in Fig. 2. Firstly, a virtual camera system is used to construct the visible and missing regions of the data. After multi-resolution downsampling, the point cloud features are extracted using MRE, and the features encoded by the multi-scale generator are decoded by the discriminator to achieve the prediction and completion of the missing regions. Finally, a loss is established by the dual-discriminator and the generator is optimized through adversarial training to improve the accuracy of the generator's prediction results.

**Fig. 2:**
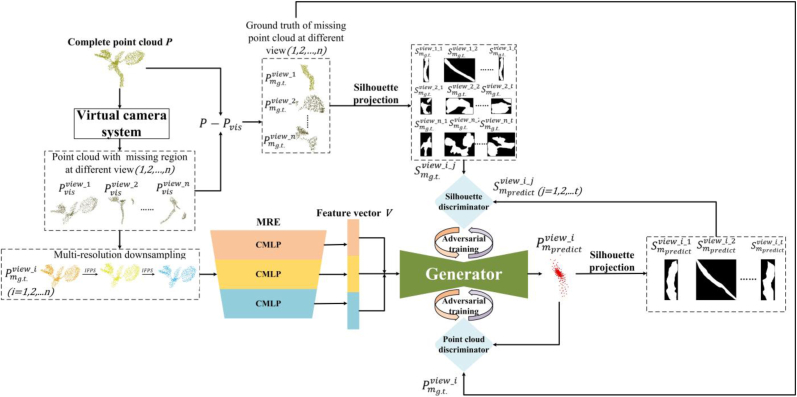
The model frame of MSDDG. With processing the input point cloud through constructing virtual camera system at different view i(i=1,2,…,n), the visible point cloud $P_{vis}$ and missing region point cloud $P_{m_{g.t.}}$ which are used to generate projection silhouette S from complete point cloud p are obtained. S is specifically defined as the projected silhouette images under different multi-view projection views j(j=1,2,…,t). Following this, the generator, getting adversarial training from dual-discriminators, utilizes feature vector V which is gained from the multi-resolution encoder to predict missing region point cloud $P_{m_{pred}}$ at different view.

#### 2.2.1 Virtual camera system

The network constructed in this study takes the complete point cloud P of a sample as its input during training. It should be emphasized that the virtual camera projection module is designed as an auxiliary data preprocessing and training constraint mechanism, and is exclusively used in the training stage. During inference, the network directly predicts the missing region without invoking the projection module, thereby avoiding additional memory overhead and computational cost in practical deployment. The size of P is N×3, where N represents the number of points in the point cloud. The network predicts the missing region's point cloud based on the point cloud of the visible area and trains it against the actual missing region's point cloud as the reference value. To obtain the point cloud visible region and missing region that conform to the optical occlusion relationship in the perspective imaging relationship, a point cloud data pre-processing process of constructing a virtual camera is proposed. For each complete point cloud, different visible regions and corresponding missing region and their projection silhouette images are obtained at multi-view projection views.

As shown in Fig. 3, based on the position of the camera during data acquisition, a spherical planar space is constructed with the center of the point cloud as the sphere's center and the radius equal to the distance from the virtual camera position to the point cloud center. The distribution of the plant point cloud data range lies within this spherical planar space. By establishing an observation plane with the viewing direction as the normal vector, the projection silhouette images of the point cloud from different viewing angles are obtained, and the occlusion relationships of points are determined. This allows the segmentation of the complete point cloud into visible and missing regions. Assuming the point cloud has N points, the coordinates of the center point $p_{cen}$ can be obtained by averaging the coordinates of all points in the point cloud using the following formula:

(8) $$p_{cen}=\left(x_{cen},y_{cen},z_{cen}\right)=\frac{1}{N}\sum_{i=1}^{N}p_{i}$$

where, $\left(x_{cen},y_{cen},z_{cen}\right)$ represent the coordinates of the point cloud center. N is the number of points contained in the point cloud, and $p_{i}$ denotes the coordinates (x,y,z) of each point in the point cloud. The position of the virtual camera is chosen on the spherical surface centered at the point cloud center, meaning any point on this sphere can serve as a viewpoint. From the viewpoint to the point cloud center, a projection plane is established, whose normal vector satisfies:

(9) $$\vec{n}\cdot \left(p_{view}-p_{cen}\right)=0$$

where $p_{view}$ is an arbitrarily selected viewpoint on the spherical surface, and $\vec{n}$ denotes the normal vector of the projection plane established from the viewpoint to the point cloud center:

(10) $$\vec{n}=p_{cen}-p_{view}$$

In the point cloud coordinate system, a projection plane is established. Combined with the viewing direction—that is, the projection relationship from the point cloud points to the projection plane—a projected silhouette image of the point cloud under the corresponding viewpoint can be generated. The position $p_{proj}$ of a point on the projection plane is calculated as follows:

(11) $$p_{proj}=p_{i}-\frac{\vec{n}\cdot \left(p_{i}-p_{cen}\right)}{{\left\|\vec{n}\right\|}^{2}}\cdot \vec{n}$$

**Fig. 3:**
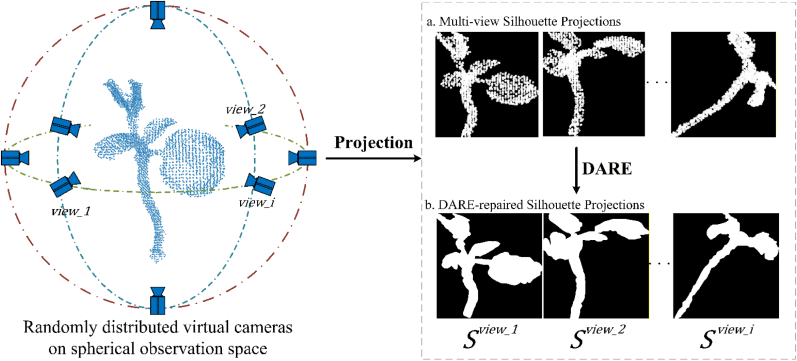
Obtaining visible and occluded region of a point cloud in different view point by constructing virtual camera. a. silhouette of point cloud in different view, b. the hole pixels in the projection map are filled using DARE.

As shown in Fig. 3a, the 2D point density of a fixed-size point cloud projected from different viewpoints varies significantly. In such cases, the pixel radius used to render the projected silhouette image may fail to cover the visible surface region, resulting in the appearance of “hole pixels.” To address this issue, this study adopts Density-Aware Radius Estimation (DARE) to determine an appropriate pixel radius for each point under different viewpoints—reducing the radius in high-density areas and increasing it in low-density areas [40].

Initially, a predefined pixel radius is assigned to render the points onto the projection image, and this radius should be large enough to cover the projection area. Let A denotes the number of visible pixels in the projection image, and M denotes the total number of projected points under the current viewpoint. The point density d in the projection image can then be calculated as follows:

(12) d=M/A

An inverse proportional function is used to model the relationship between the actual point radius r and the point density d, with a scaling factor η introduced:

(13) r=η/d

For each category of projected points, the density can be approximated using the average number of visible pixels across all projection images in the dataset. In this way, as illustrated in Fig. 3b, the pixel radius of the point cloud can be dynamically adjusted based on the point density, resulting in smoother and hole-free projected silhouette images. Using the above method, viewpoints are randomly selected on the spherical surface. For each viewpoint, a projection silhouette image is rendered, and the occlusion relationships of the points are determined through the projection plane, enabling the segmentation of visible and missing regions of the point cloud under the current viewpoint.

Using the above method, viewpoints are randomly selected on the spherical surface. For each viewpoint, a projection silhouette image is rendered, and the occlusion relationships of the points are determined through the projection plane, enabling the segmentation of visible and missing regions of the point cloud under the current viewpoint.

The core regulatory function of the multi-view silhouette projection module is to impose geometric consistency constraints on the generated point cloud before loss calculation. After the generator outputs the predicted point cloud of the missing region, we first perform multi-view projection on the generated point cloud to obtain its corresponding silhouette maps under preset virtual viewpoints, and align them with the ground-truth multi-view silhouettes of the complete plant. 3D points whose projected positions fall outside the ground-truth silhouette boundaries are determined as erroneously generated points that do not conform to the real plant morphology, and are directly zeroed out and eliminated from the effective computation flow before the discriminator input and loss function construction. This mechanism not only forces the generator to learn biologically plausible contour specifications, but also reduces invalid tensor computation and gradient cache occupation, as the eliminated points do not participate in subsequent forward calculation and back-propagation.

#### 2.2.2 Multi-resolution encoder

The accuracy of predicting missing parts depends on the encoder's ability to represent the input point cloud. Existing methods primarily use multilayer perceptions (MLPs) operating at a single resolution, limiting their capacity to capture multi-scale features.

Here, we employ a multi-resolution encoder (MRE) based on the CMLP algorithm to extract features from point clouds at multiple resolutions (Fig. 4). Unlike standard MLPs whose performance relies heavily on the max-pooling dimension, CMLP aggregates features across layers. Specifically, each point is encoded by an MLP with layers [64,128,256,512,1024], followed by max pooling on the last four layers to produce feature vectors $f_{i}$ of sizes (128,256,512,1024) for i=1,2,3,4. These are concatenated into a latent feature vector F of dimension 1920.

**Fig. 4:**
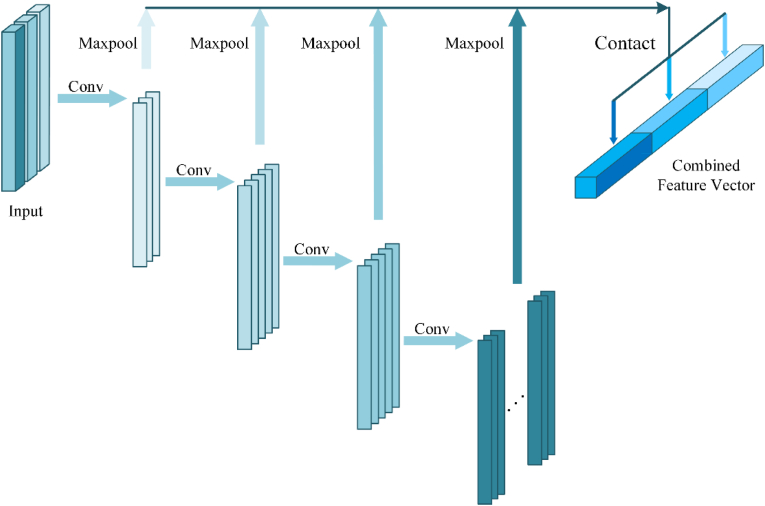
MRE is composed of CMLP. CMLP maps the combined latent vector by concatenating multiple dimensional latent vectors derived from the last four layers. It can make better use of the high-level and low-level features.

Thanks to this multi-resolution design, CMLP effectively integrates global and local information, outperforming traditional single-layer MLPs. Each CMLP layer applies convolution, batch normalization, ReLU activation, and max pooling sequentially.

The MRE processes an unordered point cloud of size N×3 through three parallel CMLPs, each extracting multi-scale features. Their outputs are concatenated and passed through a final MLP to generate a 1920-dimensional feature vector V that captures the global structure and local details of the plant point cloud.

#### 2.2.3 Multi-scale point cloud generator

The generator MSPG takes the feature vector V obtained from multi-resolution encoding by the MRE as input, and predicts the missing part of the point cloud, outputting a point cloud of fixed size M×3, where M uniformly set to 512 in this study is the number of points in the missing region $p_{m_{g.t.}}^{view\_i}$. The structure of MSPG is shown in Fig. 5. The setting of M=512 is highly matched with the input scale of the network: all input complete plant point clouds are uniformly down-sampled to 2048 points, and the point cloud scale of the visible region under single-view occlusion is about 1536 points, which can fully express the global geometric structure and local organ morphology of the experimental juvenile plants. The fixed M=512 is highly consistent with the actual missing scale in the single-view acquisition scene using sensor, and forms a reasonable proportional balance with the input visible region point number, avoiding training instability caused by input-output dimension imbalance.

**Fig. 5:**
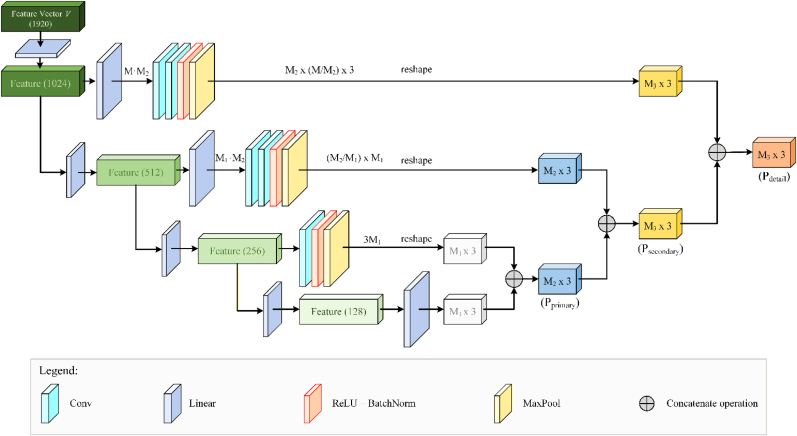
The structure of generator Multi-Scale Point Cloud Generator(MSPG). The input feature vector V sequentially passes through four fully-connected layers (FC1–FC4), where each branch generates point features with different dimensionality. FC1 and FC2 apply additional convolution and reshape operations to produce fine- and mid-level geometric features, while FC3 and FC4 yield coarse-scale features. Multi-scale features are combined via concatenate operations (⊕) to produce the primary point set P_primary_, the secondary point set P_secondary_, and finally the detailed point set P_detail_, enabling coarse-to-fine point cloud generation.

Inspired by the skip-connection mechanisms of neural network U-Net [41] and U-Net++ [42], the generator uses a multi-scale hierarchical structure with dense skip connections to capture local and global geometric features effectively. Unlike image-based CNNs, it employs fully connected layers on feature vectors, enabling flexible multi-level feature fusion.

Specifically, V is mapped through fully connected layers into four feature layers with dimensions (1024,512,256,128), corresponding to Layers 1 to 4. Lower-resolution point clouds are generated from Layers 4 and 3, concatenated to form mid-resolution outputs. Layer 2 is further processed to produce higher-resolution predictions, which are combined with earlier outputs. Finally, Layer 1 refines the point cloud to the highest resolution M×3, representing the completed missing region.

It should be noted that M, the number of predicted points for each missing region, is fixed during training and determined by the network configuration. As the network is trained with a fixed input-output scheme, it does not adaptively change M. This design is similar in nature to classification networks and ensures consistent point cloud density. Although this could theoretically lead to over-densification in some cases, the hierarchical decoding and multi-resolution loss effectively guide the distribution of generated points, preventing excessive clustering in any local region.

This hierarchical decoding, combined with skip connections, allows high-level semantic features to guide detailed local geometry reconstruction, resulting in more accurate and detailed point cloud completion.

From the perspective of framework design, the MSDDG architecture has good scalability. In practical applications, users can set different M values by adjusting the dimension of the output layer according to the target plant scale and required point cloud density, to achieve completion effects with different densities, without major modifications to the core architecture and training logic.

#### 2.2.4 Dual-discriminator

Based on the architecture of a dual-discriminator generative adversarial network, MSDDG incorporates both a spatial distance discriminator and a multi-view projected silhouette discriminator to perform adversarial training of the generator. As illustrated in Fig. 6, these two discriminators share a similar structure, with the main difference being the dimensionality of their input—either 2D projected silhouette image or 3D point cloud. The 2D projection or 3D point cloud is fed into the corresponding discriminator, which extracts features and ultimately outputs a scalar value indicating whether the input is real or fake, formulated as a binary classification problem.

**Fig. 6:**
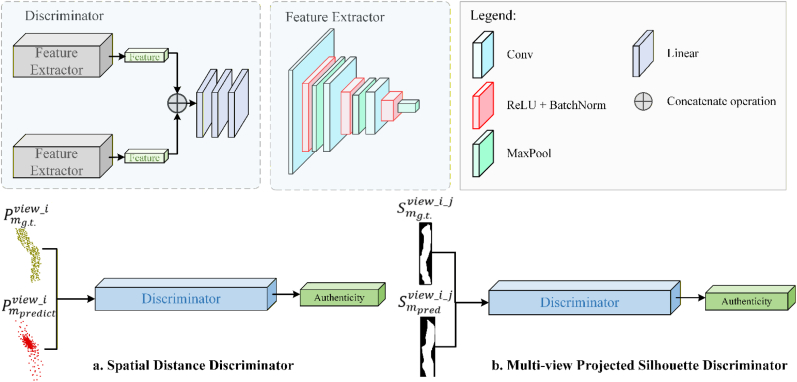
The structure of discriminator. **a**. spatial distance discriminator, **b**. multi-view projected silhouette discriminator. $view_{i}$ represents i th view point. $P_{m_{g.t.}}^{view\_i}$ and $P_{m_{pred}}^{view\_i}$ denote the ground truth and predicted value of point cloud of missing region respectively. Meanwhile, $S_{m_{g.t.}}^{view\_i\_j}$ and $S_{m_{pred}}^{view\_i\_j}$ denote projection silhouette in i th view point under j th virtual projection direction of ground truth and predicted value of point cloud.

The discriminator consists of three convolutional layers with input–output channel dimensions of [1→64],[64→128],[128→256] , each followed by ReLU activation and batch normalization. Max pooling aggregates features across scales, producing a multi-scale feature representation. This is then passed through fully connected layers to output the final authenticity score.

Although the two discriminators optimize complementary objectives—3D spatial consistency and 2D projection plausibility—potential conflicts may arise when the gradients from these losses point in different directions in the parameter space. To mitigate such conflicts, MSDDG employs alternating updates: for each iteration, the discriminators are updated first to distinguish real and generated samples, followed by generator updates using a combined multi-resolution completion loss and adversarial feedback. The dual-discriminator framework forms complementary adversarial supervision to mitigate Visual Hull ambiguity: the spatial distance discriminator focuses on the authenticity of the 3D point cloud distribution in Euclidean space, ensuring the correctness of global topology and local geometric plausibility; while the multi-view projected silhouette discriminator only supplements auxiliary constraints on multi-view contour consistency. This collaborative design ensures that the generator will not sacrifice 3D topological correctness to fit specific projection contours, and always maintains the biological rationality of the generated plant structure. This sequential update strategy reduces the risk of gradient interference, maintaining a stable training trajectory and ensuring that the generator gradually satisfies both spatial and projection-level constraints.

#### 2.2.5 Loss function

To train and update the network parameters by measuring the similarity between the predicted point cloud of the missing region $P_{m_{pred}}^{view\_i}$ and the ground truth point cloud $P_{m_{g.t.}}^{view\_i}$, our MSDDG network's loss function consists of two parts: the multi-resolution completion loss and the adversarial loss. The multi-resolution completion loss measures the discrepancy between the predicted and ground truth point clouds at different resolutions of the missing region. The adversarial loss optimizes the MRE and the generator to make the predicted point clouds more consistent with their true shapes.

To simplify the description, the predicted missing point cloud at the view_i perspective, $P_{m_{pred}}^{view\_i}$, corresponds to $P_{\det}$, and the ground truth point cloud $P_{m_{g.t.}}^{view\_i}$ corresponds to $P_{g.t}$; both have a size of M×3. The MSDDG, which includes the multi-resolution encoder (MRE), multi-scale generator, spaatial distance discriminator, and projected silhouette discriminator, is trained in a supervised manner through a multi-objective loss function. The multi-objective loss function is defined as follows:

(14) $$L=\lambda_{com}L_{com}+\lambda_{dis}L_{dis}+\lambda_{sil}L_{sil}$$

where $\lambda_{com},\lambda_{dis},\lambda_{sil}$ are the weights of $L_{com},L_{dis},L_{sil}$ in the multi-objective loss function, respectively. $L_{com}$ represents the Chamfer Distance (CD) loss of point clouds at three different resolutions as the multi-resolution completion loss. $L_{dis}$ and $L_{sil}$ denote the binary cross-entropy losses between the predicted point cloud and the ground truth point cloud, and between the predicted point cloud and the multi-view projected silhouettes, respectively. The details are as follows:

(15) $$L_{com}=d_{{CD}_{1}}\left(P_{\det},P_{g.t.}\right)+\alpha d_{{CD}_{2}}\left(P_{\sec},P_{g.t.}^{1}\right)+\beta d_{{CD}_{3}}\left(P_{pri},P_{g.t.}^{2}\right)$$

(16) $$L_{dis}=\sum_{1\leq i\leq N_{Set}}\sum_{1\leq i\leq N_{Set}}\log\left(D_{dis}\left(P_{y_{i}}\right)\right)+\sum_{1\leq j\leq N_{Set}}\log\left(1-D_{dis}\left(G\left(P_{x_{j}}\right)\right)\right)$$

(17) $$L_{sil}=\frac{1}{N_{proj}}\sum_{v_{proj}=1}^{N_{proj}}L_{proj}$$

where, $P_{\det},P_{\sec},P_{pri}$ represent the generated high, medium, and low resolution point clouds, respectively. α and β denote the weights of the Chamfer Distance (CD) loss at the medium and low resolutions. $P_{g.t.}^{1}$ and $P_{g.t.}^{2}$ refer to the medium and low resolution ground truth point clouds obtained by applying one and two iterations of iterative farthest point sampling (IFPS) on the ground truth point cloud $P_{g.t.}$, respectively. $d_{{CD}_{1}}$, $d_{{CD}_{2}}$, and $d_{{CD}_{3}}$ represent the Chamfer Distance losses between two point clouds at different resolutions. $D_{dis}\left(\right)$ denotes the spatial distance discriminator, and G() denotes the multi-scale generator. $P_{x_{j}}$ is the visible region point cloud $P_{vis}^{view\_i}$ fed into the network, and $P_{y_{i}}$ is the ground truth point cloud of the missing region $P_{m_{g.t.}}^{view\_i}$. $N_{Set}$ denotes the batch size of the dataset. $L_{sil}$ represents the binary cross-entropy loss between the projected silhouette image of the predicted point cloud and the ground truth from view proj in the virtual projection space. The definitions of $d_{CD}$ and $L_{proj}$ are given as follows:

(18) $$d_{CD}\left(Set_{1},Set_{2}\right)=\frac{1}{N_{Set_{1}}}\sum_{p_{x}\in Set_{1}}\min_{p_{y}\in Set_{2}}{\left\|p_{x}-p_{y}\right\|}_{2}^{2}+\frac{1}{N_{Set_{2}}}\sum_{p_{y}\in Set_{2}}\min_{p_{x}\in Set_{1}}{\left\|p_{y}-p_{x}\right\|}_{2}^{2}$$

(19) $$L_{proj}=\sum_{1\leq i\leq S}\log\left(D_{sil}\left(S_{proj}\left(P_{y_{i}}\right)\right)\right)+\sum_{1\leq j\leq S}\log\left(1-D_{sil}\left(S_{proj}\left(P_{x_{i}}\right)\right)\right)$$

where, $N_{Set_{1}},N_{Set_{2}}$ represent the predicted and ground truth point clouds of the missing region, respectively. $p_{x}$ and $p_{y}$ are individual points belonging to $Set_{1}$ and $Set_{2}$, respectively. $D_{sil}\left(\right)$ denotes the silhouette discriminator, and $S_{proj}$ represents the projected silhouette image of the point cloud from view proj in the virtual projection space.

During network training and weight updates, the adaptive learning rate optimization method Adam is used to dynamically adjust the learning rate for updating the model parameters and optimizing the generator toward convergence.

The combination of these losses introduces a multi-objective optimization problem. While the generator receives gradients from all three terms, oscillations in $L_{dis}$ and $L_{sil}$ may occur due to adversarial dynamics. However, empirical observation shows that these oscillations remain bounded, and the alternating optimization ensures that the generator updates progressively satisfy both discriminators. This implies a dynamic equilibrium in which the generator improves reconstruction fidelity while adhering to adversarial supervision without destabilizing convergence.

During training, the generator and discriminators are optimized alternately: the discriminators are updated to distinguish real from generated samples, and then the generator is updated using the combined loss. While $L_{dis}$ and $L_{sil}$ may oscillate due to adversarial dynamics, these oscillations remain bounded, and alternating updates ensure stable convergence. $L_{sil}$ aggregates supervision across multiple projections, reducing the risk of fragmented artifacts from Visual Hull ambiguity and encouraging coherent 3D structure reconstruction.

### 2.3 Evaluation metrics

To evaluate the performance of completed point cloud, four evaluation metrics including Chamfer Distance(CD), Unidirectional Chamfer Distance(UCD), Hausdorff Distance(HD) and F1-score are used to measure the set-level distance and similarity between the predicted point cloud of the ground truth. Completed point set and the ground truth is defined as $Set_{1}$, $Set_{2}$. $P_{1}$ and $P_{2}$ are respectively points belonging to $Set_{1}$ and $Set_{2}$. The Chamfer Distance is defined as equation:

(20) $$d_{CD}\left(Set_{1},Set_{2}\right)=\frac{1}{N_{Set_{1}}}\sum_{P_{1}\in Set_{1}}\min_{P_{2}\in Set_{2}}{\left\|P_{1}-P_{2}\right\|}_{2}^{2}+\frac{1}{N_{Set_{2}}}\sum_{P_{2}\in Set_{2}}\min_{P_{1}\in Set_{1}}{\left\|P_{2}-P_{1}\right\|}_{2}^{2}$$

Similar to Chamfer Distance, UCD is also an indicator used to measure the similarity between two point sets. However, it only measures the average of the mutual distances from one point set to another point set, as defined below:

(21) $$d_{UCD}\left(Set_{1},Set_{2}\right)=\frac{1}{\left|Set_{1}\right|}\sum_{P_{1}\in Set_{1}}\min_{P_{2}\in Set_{2}}{\left\|P_{1}-P_{2}\right\|}_{2}$$

The Hausdorff Distance (HD) measures the distance between two subsets in the metric space $R^{3}$. It is defined as the greatest distance from a point in one set to the nearest point in the other set. In other words, it represents the maximum of the minimum distances from points in one set to the other. The definition of HD is as follows:

(22) $$d_{HD}\left(Set_{1},Set_{2}\right)=\max\left\{\max_{P_{1}\in Set_{1}}\min_{P_{2}\in Set_{2}}\left\|P_{1}-P_{2}\right\|,\max_{P_{2}\in Set_{2}}\min_{P_{1}\in Set_{1}}\left\|P_{2}-P_{1}\right\|\right\}$$

F1-score measures the harmonic mean of precision and recall between two point sets under a matching threshold τ. A point is considered correctly matched if the nearest-neighbor distance between the two point clouds is below τ. The F1-score is the harmonic mean of Precision and Recall. It is defined as:

(23) $$F_{1}=\frac{2\cdot Precision\cdot Recall}{Precision+Recall}$$

where Precision measures the fraction of predicted points whose nearest neighbor in the ground-truth point set is within the threshold τ, and Recall measures the fraction of ground-truth points whose nearest neighbor in the predicted point set is within τ.

(24) $$Precision=\frac{1}{N}\sum_{i=1}^{N}1\left(\min_{P_{1}\in Set_{1}}{\left\|P_{1}-P_{2}\right\|}_{2}<\tau \right)$$

(25) $$Recall=\frac{1}{M}\sum_{j=1}^{M}1\left(\min_{P_{2}\in Set_{2}}{\left\|P_{2}-P_{1}\right\|}_{2}<\tau \right)$$

where, 1(·) is the indicator function that outputs 1 if the condition is satisfied and 0 otherwise, and τ is the distance threshold used to determine whether a point is considered correctly matched.

## 3 Results

### 3.1 Implementation details

#### 3.1.1 Dataset

To meet the need for high-quality training data in this study, we constructed a dataset named Plant4L for plant point cloud completion based on an existing 3D reconstruction system in our lab. Unlike previously released datasets such as PCN [17] and ShapeNet40 [43], which cover a wide range of object categories, or studies focusing on a single plant species [26], Plant4L includes four distinct plant species — sunflower, pumpkin, eggplant, and luffa — each exhibiting unique morphological complexity. All species are jointly used to train a single unified model rather than training separate models for each crop type. This mixed-species training strategy encourages the network to learn generalizable structural representations instead of species-specific geometric patterns, enabling the model to capture shared geometric priors across different plant morphologies, including variations in leaf arrangement, curvature, and stem proportion.

The dataset supports preprocessing steps for generating incomplete point clouds and projection silhouette images before training. Each input sample is down-sampled to a point cloud with 2048 points, suitable for training multi-view point cloud completion models. Each complete point cloud in the dataset is created by collecting raw point clouds, clustering the points, and applying non-rigid transformations to selected categories for data augmentation. Each complete point cloud contains over 5000 points, and the figure illustrates examples of non-rigid transformations applied to three randomly selected clusters within a plant point cloud. The dataset consists of a total of 560 sunflower, 410 pumpkin, 540 eggplant, and 390 luffa samples. Similar to other public datasets, our dataset is split into training, testing, and validation sets in a 7:2:1 ratio, resulting in 1,330, 380, and 190 samples respectively. To ensure balanced training across species, the dataset is constructed with similar sample sizes per species after augmentation: sunflower (560 samples), pumpkin (410), eggplant (540), and luffa (390). During training, samples from all species are randomly shuffled within each mini-batch to avoid species bias, and no class imbalance mitigation (e.g., weighted loss) is required [44].

#### 3.1.2 Model training

We use PyTorch as the experimental platform for MSDDG. During training, samples from all four plant species were randomly mixed within each mini-batch. No species labels or crop-specific parameters were introduced into the network. The model therefore learns shared geometric priors across different plant morphologies, including variations in leaf arrangement, curvature, and stem proportion. This design aims to improve structural generalization rather than fitting a single crop category. The network is trained using the AdamW optimizer [40] with a base learning rate of 0.0005 and a weight decay rate of 0.0001. A StepLR learning rate scheduler with fixed step size is applied to dynamically adjust the learning rate during training. All network models are deployed on an NVIDIA RTX 3090 GPU (24GB memory), with a batch size of 16 and trained for 900 epochs.

In our experiments, the dataset is split into training, testing, and validation sets in a 7:2:1 ratio. All point cloud coordinates in the dataset are normalized to the range [−1, 1].

To comprehensively evaluate the convergence behavior of MSDDG, Fig. S3 illustrates the convergence curves of these loss components across 900 epochs, including the multi-resolution completion loss $L_{com}$, the spatial discriminator loss $L_{dis}$, the silhouette discriminator loss $L_{sil}$, and the total multi-objective loss. To evaluate the convergence behavior of the proposed framework, iteration-level losses were recorded during training. Due to the adversarial nature of the optimization, high-frequency oscillations are observed in discriminator-related loss. To better reveal the underlying convergence trend, an exponential moving average (EMA) smoothing strategy was applied:

(26) $${EMA}_{t}=\alpha {EMA}_{t-1}+\left(1-\alpha \right)x_{t}$$

where $x_{t}$ denotes the loss value at iteration t, and α is seta to 0.2. This operation suppresses short-term fluctuations while preserving long-term convergence characteristics.

The multi-resolution completion loss $L_{com}$ decreases rapidly during the early training stage, indicating that the generator quickly captures coarse geometric structures of the missing regions. As training progresses, the decline becomes gradual, reflecting refinement of local geometric details. The spatial discriminator loss $L_{dis}$ and silhouette discriminator loss $L_{sil}$ exhibit moderate oscillations throughout training, which are typical characteristics of adversarial learning. Importantly, the oscillations remain bounded and do not amplify over time, suggesting that the generator and discriminators gradually reach a dynamic equilibrium. The total loss follows a consistent downward trend with limited fluctuations. Although a temporary spike occurs around epoch 300, the model quickly recovers and continues converging. This behavior indicates that the dual-discriminator framework does not destabilize optimization and that the training process remains well-controlled.

Overall, the simultaneous convergence of all four loss components demonstrates stable adversarial training and confirms that the proposed multi-scale dual-discriminator architecture achieves balanced optimization between geometric reconstruction and adversarial supervision.

### 3.2 Point cloud completion result

#### 3.2.1 The completion results

To provide a clear demonstration of the point cloud completion results, a pumpkin specimen from the dataset was chosen for visualization, as presented in Fig. 7. The completion results for each plant category were quantitatively evaluated by average metrics shown in Table 1. The visualization results demonstrate that the model successfully completes plant point clouds of different species with varying shapes and bending degrees under different viewpoints. The completion results indicate that the proposed network effectively completes missing regions in the point clouds of various plant categories in the dataset. The completion process successfully recovers internal and edge points of leaves and stems occluded due to viewpoint occlusion, showing strong point cloud completion ability. By comparing VMV-1 (where the missing region spatial distribution is continuous) with other views, it is evident that when the missing region is spatially concentrated, the completion is better, and no outlier points outside the plant point cloud range appear. For VMV-2 and VMV-3, the missing regions are more dispersed over the plant, with larger missing areas. Although completion is achieved in these regions, significant outliers appear in the completed point clouds, resulting in shapes inconsistent with the actual plant, where generated points tend to cluster in specific missing regions.

**Fig. 7:**
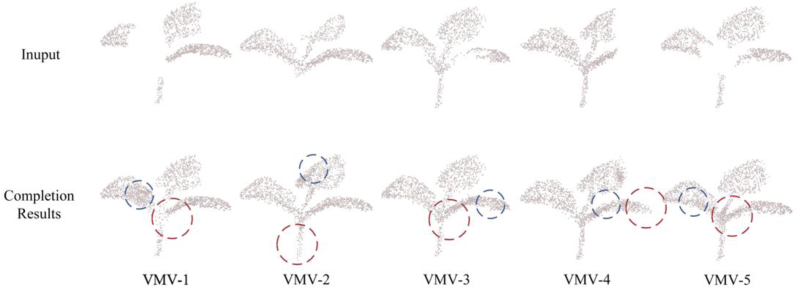
The visualization of the plant point cloud completion for Plant4L(Pumpkin). VMV-i represents the representative Virtual Multi-View(i=1,2,3,4,5), which adheres to the above-mentioned virtual camera system for partitioning the different inputs. The method we propose can complete the localized detailed parts of plants such as leaf and stems, which are closest to Reference Point Cloud.

**Table 1:** The average evaluation metrics compared with other methods on the Plant4L dataset. The best result is highlighted.

Method	Average			Pumpkin			Sunflower			Eggplant			Luffa		
	$d_{CD}$	$d_{UHD}$	$F_{1}$	$d_{CD}$	$d_{UHD}$	$F_{1}$	$d_{CD}$	$d_{UHD}$	$F_{1}$	$d_{CD}$	$d_{UHD}$	$F_{1}$	$d_{CD}$	$d_{UHD}$	$F_{1}$
PF-Net	0.699	1.069	0.540	0.349	0.966	0.287	1.186	1.412	0.015	0.562	1.102	0.166	0.699	0.797	0.072
FoldingNet	0.805	1.084	0.062	0.503	0.847	0.126	0.867	1.071	0.002	0.835	0.899	0.064	1.014	1.519	0.054
SA-Net	0.487	0.824	0.248	0.328	0.695	0.303	0.287	0.978	0.377	0.342	0.835	0.309	0.991	0.789	0.001
**MSDDG(Ours)**	**0.187**	**0.309**	**0.906**	**0.211**	**0.439**	**0.903**	**0.209**	**0.362**	**0.916**	**0.126**	**0.361**	**0.853**	**0.200**	**0.354**	**0.957**

To evaluate the plant point cloud completion performance, representative plant point clouds were selected from the validation set of the dataset constructed in this study, which contains four plant species. The point clouds were visualized in MeshLab software to observe the generation quality of missing or invisible regions. In addition to non-rigid transformations, to assess the robustness of the model against orientation variations, we conducted a small-scale rotation experiment. Input point clouds were rotated around Z axes with angles of 30°, 60°, and 90° before being fed into the network. All point clouds were normalized to the [−1,1] range prior to rotation. And the results indicated that minor rotations caused negligible differences in completion accuracy, demonstrating the model's invariance to moderate changes in point cloud orientation. The result is shown in Table S1.

For each category, we selected the most representative plant point cloud as the reference point cloud as shown in Fig. S4. To avoid the incomplete issues caused by the visualization of point cloud and present more details, we selected the point cloud completion result of a plant and observed it from three different views, corresponding to the front, side and top views as shown in Fig. S5.

By simulating a virtual camera in the point cloud preprocessing step, five virtual viewpoints were randomly selected on the virtual observation sphere surface to mimic data loss caused by optical imaging from different perspectives. For each virtual viewpoint, the plant point cloud was divided into visible and missing regions, enabling assessment of completion performance under five virtual observation views for each plant species in the validation set. Based on this, we applied the multi-view data partitioning strategy to examine the completion performance under various patterns of missing regions. Fig. S6, Fig. S7 and Fig. S8 presents the visualization results of point cloud completion for other 3 categories in Plant4L.

#### 3.2.2 Comparison analysis

The completion results and evaluation metrics of the proposed MSDDG on the validation set have been described in detail in Section 3.2.1. We selected three representative point cloud completion methods—PF-Net, FoldingNet, and SA-Net. To ensure a fair comparison and highlight the differences among these methods, we selected identical plant instances and defined consistent missing regions under the same viewpoints. In this setting, Pumpkin, Eggplant, and Luffa represent cases with spatially continuous missing regions, while Sunflower exhibits dispersed missing areas. All methods were evaluated on the same training/testing sets and using the same evaluation metrics.

Table 1 presents a quantitative comparison of our proposed method MSDDG against the other methods on the Plant4L dataset. Fig S 9 shows the qualitative visualization results of point cloud completion on the validation set of Plant4L. In the visualizations, the brown points represent the incomplete input point clouds, the blue points denote the ground truth of the missing regions, and the red points indicate the predicted completions.

For PF-Net, the network successfully completed one missing region of the Pumpkin plant. However, it failed to reconstruct another missing leaf within the same area, and some of the generated points extended beyond the actual leaf structure, particularly at the leaf tip. In the Sunflower case, which involves spatially dispersed missing regions, PF-Net primarily reconstructed the missing stem, while ignoring the absent leaf areas. In the more structurally complex plants such as Eggplant and Luffa, although PF-Net generated predictions, most of the completed points were misaligned with the actual missing regions, and several predicted points extended beyond the original plant boundary.

FoldingNet demonstrated better performance in the Pumpkin case, successfully completing the missing regions with a relatively even distribution of points. However, a portion of the completed points still deviated beyond the original plant shape. For the other three plant types, FoldingNet failed to produce satisfactory completions; the predicted points were either scattered away from the true missing regions or overly concentrated in a limited area, reducing reconstruction accuracy.

Regarding SA-Net, it preserved the original leaf contours while completing the missing regions of the Pumpkin plant without producing overshooting points. Nonetheless, it failed to reconstruct the missing stem segment. In the Sunflower case, while the overall shape of the predicted point cloud resembled the ground truth, significant spatial deviations were observed, resulting in completions that did not align with the actual missing areas.

In comparison, our proposed MSDDG achieves significantly more accurate and structurally consistent completions across all plant categories. For both spatially continuous and dispersed missing regions, MSDDG is able to generate point clouds that closely match the original plant morphology, without producing excessive points beyond structural boundaries or neglecting specific missing parts. The results demonstrate that MSDDG exhibits better spatial alignment, improved completeness, and stronger adaptability to diverse plant structures compared to existing methods.

### 3.3 Ablation studies

#### 3.3.1 Model design analysis

To evaluate the contribution of the proposed components, we analyzed the specific impact of three modules on the performance of the newly developed method. This analysis focuses on how each module enhances the overall performance, as measured by three key metrics: $d_{CD}$, $d_{UCD}$, $d_{HD}$ and F-Score($F_{1}$). The quantitative results are summarized in Table 2**,** and the qualitative comparisons across four plant categories are visualized in Fig. S10**.**

**Table 2:** Ablation experiments of the proposed modules. MLP denotes a baseline feature extraction module composed exclusively of multiple fully connected layers. MRE extends the MLP architecture by incorporating feature concatenation operations to enhance representational capacity. MRD represents a multi-resolution reconstruction decoder that progressively generates point clouds across hierarchical resolutions. MSPG refers to a multi-scale point generator that adopts a hierarchical architecture with dense skip connections to synthesize point clouds at multiple granularities. S-D denotes the multi-view silhouette discriminator employed within the dual-discriminator framework.

Method					Configuration	$d_{CD}$	$d_{UCD}$	$d_{HD}$	$F_{1}$
MLP	MRE	MRD	MSPG	S-D					
✓		✓			Baseline	0.611	0.423	0.923	0.022
**✓**		**✓**		**✓**	+S-D	0.573	0.384	0.912	0.138
**✓**	**✓**	**✓**			+MRE	0.789	0.289	1.209	0.005
**✓**	**✓**		**✓**		+MRE + MSPCG	0.505	0.327	0.874	0.193
**✓**	**✓**		**✓**	**✓**	+ MRE + MSPCG + S-D	**0.187**	**0.309**	**0.462**	**0.906**

The baseline model relying solely on an MLP encoder–decoder shows limited ability to recover missing structures, resulting in relatively high errors ($d_{CD}=0.611$, $d_{UCD}=0.423$) and obvious structural gaps. Introducing the S-D module improves global shape plausibility by enforcing silhouette-level constraints, yielding reduced $d_{CD}$ (0.573) and a clear increase in F-Score (from 0.022 to 0.138). However, its ability to handle complex occlusions remains restricted without stronger feature representations. With the addition of the MRE module, the encoder benefits from multi-level feature aggregation, providing richer representations for downstream reconstruction. The combination of MRE and MSPCG further boosts performance by enabling multi-resolution point synthesis, which improves geometric fidelity and reduces errors to $d_{CD}=0.505$. The visual results show more complete recovery of thin or fragmented structures. The full configuration, integrating MRE, MSPCG, and S-D, achieves the most significant performance gain, reaching the lowest errors ($d_{CD}=0.187$, $d_{UCD}=0.309$, $d_{HD}=0.462$) and the highest F-Score (0.906). This model recovers both global topology and detailed local geometry most effectively, while also reducing hallucinated structures across all plant categories. These results confirm that the three modules provide complementary benefits and collectively lead to the strongest reconstruction performance.

#### 3.3.2 Analysis of number of virtual multi-view projection silhouette

The number of silhouette projections used in the multi-view projected silhouette discriminator has a notable impact on its effectiveness. As shown in Table S2, using 10 projection views achieves the best performance across all metrics, providing lower distance errors and a higher F_1_ score. Fewer views lead to insufficient geometric coverage, while more views introduce redundancy without further improvement. Thus, 10 views offer the most efficient and balanced configuration for the discriminator.

### 3.4 Point cloud completion application for 3D reconstruction from single view

#### 3.4.1 Point cloud completion application

To evaluate the effectiveness of the proposed MSDDG framework in 3D reconstruction, we applied it to single-view point clouds of plant samples not included in the Plant4L dataset. Unlike traditional multi-view reconstruction methods, which require multiple scans and complex point cloud registration, our approach attempts to reconstruct the complete 3D structure directly from a single view.

Fig. 8 shows the point cloud obtained from a single view. Due to occlusions and the complex structure of the plant, large missing regions can be observed, especially in areas marked with red circles. These incomplete point clouds were processed using the pretrained MSPG module without any fine-tuning on the target plant types. Fig. 8 presents the completion results. The orange points indicate the original incomplete input, while the red points represent the predicted completions generated by the MSPG. The results show that the proposed method is capable of reconstructing major missing structures. For example, in point clouds (1) and (4), substantial structural information has been successfully recovered. In some cases, however, the predicted points tend to cluster spatially, and when the missing area is extensive—such as in the blue-circled region in (2)—the predicted geometry deviates from the actual structure. In point cloud (3), where the missing regions are scattered and distributed across the plant, the model exhibits difficulty in producing reliable completions.

**Fig. 8:**
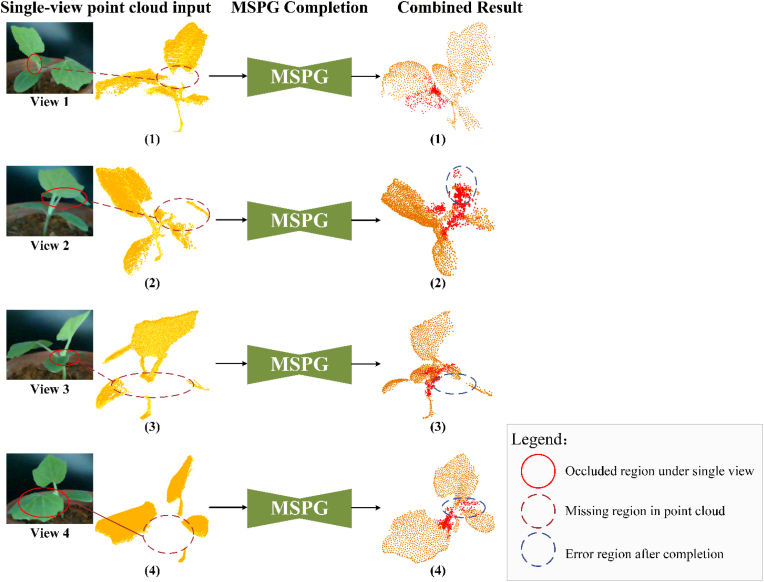
Single-view point cloud completion results using the proposed MSDDG framework. Incomplete point clouds captured from single views, with missing regions highlighted in red circles. Completion results predicted by the MSPG module; red points denote newly generated structures. Combined visualization of input (orange) and predicted (red). Completion results produced by the MSPG for each corresponding view. Blue circles indicate regions where predictions deviate significantly from the ground truth structure.

To further investigate the applicability of the proposed framework to large-scale point clouds, we conducted completion experiments on plant point clouds containing approximately 100k points. Since the pretrained model was trained with a fixed and relatively small generation parameter M=512, the number of predicted completion points remains constant during inference. As a result, when applied to high-density point clouds, the generated completion points appear comparatively sparse relative to the overall scale of the input data.

Although the predicted points are limited in quantity, they still provide a coarse estimation of the missing structures and preserve the primary geometric characteristics of the occluded regions. This behavior is consistent with the intrinsic design of the network. Essentially, the proposed model functions as a classification-based completion network, where each generated point corresponds to a predicted structural hypothesis rather than a dense geometric reconstruction. Therefore, when the input scale increases significantly without adjusting the generation capacity, the completion results may exhibit reduced density while still maintaining structural consistency.

These findings indicate that while the MSDDG framework can generalize to large-scale single-view point clouds, its completion density is constrained by the fixed output size of the pretrained model. Future work may consider adaptive point generation strategies or hierarchical refinement modules to better accommodate high-resolution reconstruction tasks.

Overall, the MSDDG-based completion approach enhances the 3D completeness of single-view reconstructions and reveals occluded regions to some extent. Nonetheless, its performance declines when applied to plant categories not seen during pretraining, particularly in cases of large or irregularly missing regions.

It should be noted that the plant samples considered in this study do not contain individuals with more than 50 leaves; therefore, extremely large and highly complex canopy structures were not involved in the current evaluation.

In practical data acquisition, the raw single-view point clouds typically contain approximately 100k points. However, prior to being fed into the completion network, the point clouds are downsampled using Iterative Farthest Point Sampling (IFPS) to match the fixed input size required by the pretrained model. This preprocessing step ensures consistency between the inference data and the training configuration, where the generation parameter M=512 is fixed.

Therefore, the sparsity observed in large-scale completion experiments is not caused by data acquisition limitations, but rather by the predefined output capacity of the generation module. The network is designed to predict a fixed number of structural hypothesis points rather than perform dense geometric reconstruction. Adjusting the generation scale or incorporating hierarchical refinement strategies may further improve reconstruction density for high-resolution plant models.

#### 3.4.2 Analysis of phenotypic parameters

Although the proposed MSDDG framework demonstrates structural recovery capability under single-view conditions, practical phenotypic analysis requires not only geometric completion but also accurate spatial calibration, scale restoration, and organ-level identification. Therefore, to further validate the applicability of the proposed framework in real phenotyping scenarios, a complete acquisition-to-measurement pipeline was established, as described below.

To evaluate the practical applicability of the proposed framework in plant phenotyping, a multi-view rotational 3D reconstruction system, which was utilized to acquire original data of plant, was constructed based on a fixed LiDAR–turntable configuration. The raw plant point clouds acquired by the LiDAR sensor are represented in metric units. To match the input configuration of the pretrained generator, the point clouds are normalized into a unit. Each point is then normalized accordingly.

The complete mathematical derivation of the coordinate transformation, normalization and inverse normalization process is as follows: First, the raw point cloud from the depth data is converted from the camera coordinate system to the world coordinate system:

(27) $$p_{w}=R_{cw}p_{c}+t_{cw}$$

where $p_{c}\in R^{3}$ is the 3D coordinate of the point extracted from the depth data in the camera coordinate system, $p_{w}\in R^{3}$ is the coordinate in the unified metric world coordinate system after conversion, $R_{cw}\in R^{3\times 3}$ and $t_{cw}\in R^{3}$ are the pre-calibrated rotation matrix and translation vector from the camera coordinate system to the world coordinate system, obtained by hand-eye calibration between the L515 sensor and the rotation platform. Then, the world coordinate point cloud is normalized to the range of [−1,1] for network input:

(28) $${\hat{p}}_{w}=2\cdot \frac{p_{w}-m_{\min}}{m_{\max}-m_{\min}}-1$$

where $m_{\min}={\left[x_{\min},y_{\min},z_{\min}\right]}^{T}$ and $m_{\max}={\left[x_{\max},y_{\max},z_{\max}\right]}^{T}$ are the minimum and maximum values of the plant point cloud in the three coordinate axes of the world coordinate system, respectively, and ${\hat{p}}_{w}\in {\left[-1,1\right]}^{3}$ is the normalized point coordinate for MSPG input. After point cloud completion, the inverse normalization is performed to restore the metric world coordinate scale:

(29) $$p_{w,pred}=\left(\frac{{\hat{p}}_{w,pred}+1}{2}\right)⨀\left(m_{\max}-m_{\min}\right)+m_{\min}$$

where ${\hat{p}}_{w,pred}$ is normalized point coordinate of the completed point cloud output by MSPG, $p_{w,pred}$ is the completed point cloud restored to the metric world coordinate system, and ⨀ denotes the Hadamard product (element-wise multiplication). This process ensures that the completed point cloud is completely consistent with the original acquisition data in terms of spatial scale and coordinate system, providing an accurate metric benchmark for subsequent phenotypic measurement.

For leaf segmentation, we adopted a 2D-3D geometrically aligned instance segmentation pipeline based on pre-calibrated camera parameters. First, the incomplete point cloud is fed into MSPG to complete the occluded regions, and the completed point cloud is restored to the world coordinate system through inverse normalization. Meanwhile, the synchronously acquired RGB image is fed into a pre-trained Mask R-CNN network to obtain pixel-level leaf instance segmentation masks, where each mask corresponds to an independent leaf in the current view. Then, based on the pre-calibrated camera intrinsic and extrinsic parameters, the completed 3D point cloud is projected to the 2D RGB image pixel plane through the pinhole camera projection model:

(30) $$\left[\begin{array}{c} u\\ v\\ 1 \end{array}\right]=K\left[R|t\right]\left[\begin{array}{c} X\\ \begin{array}{c} Y\\ \begin{array}{c} Z\\ 1 \end{array} \end{array} \end{array}\right]$$

where (X,Y,Z) is the 3D coordinate of the point in the completed point cloud under the world coordinate system, (u,v) is the pixel coordinate of the 3D point projected to the RGB image plane, K is the pre-calibrated intrinsic matrix of the L515 sensor, including the focal length $f_{x},f_{y}$ and the principal point coordinate $c_{x},c_{y}$, and [R|t] is the extrinsic matrix from the world coordinate system to the RGB image plane, which is obtained by the pre-calibration of the acquisition system. This formula establishes the strict geometric mapping relationship between each 3D point in the point cloud and the corresponding pixel in the RGB image. Then, we perform IOU matching between the projected point cloud and the leaf instance masks output by Mask R-CNN, to establish the correspondence between 2D leaf instances and 3D point cloud clusters. For the k-th leaf instance mask $M_{k}\in {\left\{0,1\right\}}^{H\times W}$ output by Mask R-CNN (where H and W are the height and width of the RGB image, and 1 indicates that the pixel belongs to the k-th leaf), the intersection over union (IOU) between the mask and the projected point cloud pixel set $P_{proj}$ is defined as:

(5) $${IOU}_{k}=\left|\frac{M_{k}\cap P_{proj}}{M_{k}\cup P_{proj}}\right|$$

where |·| denotes the number of pixels in the set. We set the IOU threshold to 0.87 in this study, and the 3D point cloud subset with the highest matching degree with the highest matching degree with the k-th leaf instance mask is screened out through the threshold, to establish a one-to-one correspondence between the 2D leaf instance and the 3D point cloud cluster. Finally, the accurate segmentation of individual leaf point clouds is completed based on the matching results:

(6) $$C_{k}=\left\{p\in P_{pred}|proj\left(p\right)\in M_{k},{IOU}_{k}\geq \tau \right\}$$

where $P_{pred}$ is the completed complete 3D point cloud, proj(·) denotes the projection operation of the pinhole camera model, τ=0.87 is the IOU matching threshold, and $C_{k}$ is the 3D point cloud cluster of the k-th leaf obtained by segmentation. This pipeline uses the pixel-level 2D semantic information to constrain the 3D point cloud segmentation, which not only avoids the over-segmentation or under-segmentation caused by only using hierarchical clustering, but also can verify the integrity of the completed region through the 2D instance mask. For the local regions that are not fully completed by MSPG, we can supplement and correct them through the geometric constraints of the 2D instance mask, to ensure the reliability of subsequent phenotypic parameter calculation. The number of leaf instances obtained by Mask R-CNN also provides a clear category basis for the hierarchical clustering of the aligned point cloud, and finally the accurate organ-level phenotypic analysis is realized. The complete implementation details of the above process have been supplemented in Section 3.4.2 of the revised manuscript, and the complete workflow is shown in Fig. 9.

**Fig. 9:**
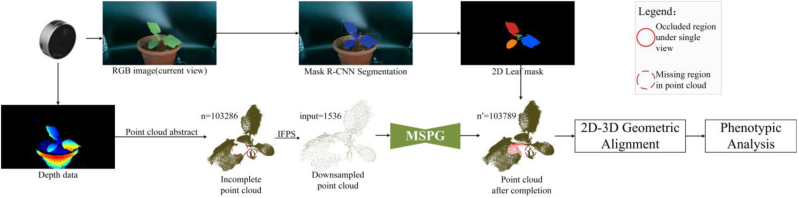
The pipeline acquires synchronous RGB-D data via Intel RealSense L515, and sequentially performs MSPG point cloud completion, Mask R-CNN leaf instance segmentation, 2D-3D geometric alignment, hierarchical clustering-based leaf separation, and organ-level phenotypic trait analysis. n=103286 and $n^{\prime }=n+512=103789$ denote the point counts of the incomplete and completed point clouds, respectively.

Although the proposed MSPG framework effectively predicts the geometric distribution of most missing regions in large-scale point clouds (approximately 100k points), a small portion of occluded areas may remain incompletely reconstructed. This limitation primarily arises from severe self-occlusion in single-view acquisition and the inherent uncertainty of generative inference under highly ambiguous structural conditions.

In this experiment, 46 individual leaf point clouds collected from three plants were used to evaluate the reliability of the proposed completion method for leaf area estimation. Leaf area was computed using a triangular mesh–based surface calculation, and the accuracy was assessed by comparing the results with manual ground-truth measurements. The correlation analyses are shown in Fig. 10.

**Fig. 10:**
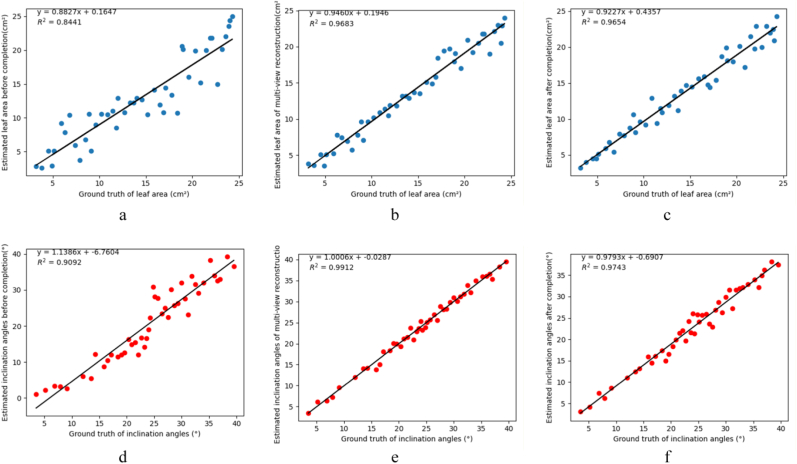
Correlation analysis between estimated leaf area and inclination angles and manual ground-truth measurements. (a) Leaf area estimates obtained from incomplete point clouds before completion. (b) Leaf area estimates derived from multi-view reconstructed point clouds. (c) Leaf area estimates after applying the proposed completion method. (d) Inclination angles obtained from incomplete point clouds before completion. (e) Inclination angles estimates derived from multi-view reconstructed point clouds. (f) Inclination angles after applying the proposed completion method.

For leaf area estimation (Fig. 10a–c), before point cloud completion (Fig. 10a), the estimated leaf area exhibited an $R^{2}$ of 0.8441, indicating that incomplete geometry led to noticeable underestimation or overestimation in some cases. After applying our completion method (Fig. 10c), the estimation accuracy improved substantially, with the $R^{2}$ increasing to 0.9654, demonstrating that geometric recovery effectively reduced structural loss and enhanced measurement reliability. For comparison, leaf areas estimated from multi-view reconstructed point clouds (Fig. 10b) achieved an $R^{2}$ of 0.9683, which is comparable to the performance achieved through our completion pipeline.

For leaf inclination angle estimation (Fig. 10d–f), before point cloud completion (Fig. 10d), the estimated inclination angles showed an $R^{2}$ of 0.9092, with significant deviations in cases where the leaf edge or surface was truncated by occlusion. After applying our completion method (Fig. 10f), the estimation accuracy improved markedly, with the $R^{2}$ rising to 0.9743, indicating that the recovery of missing leaf surfaces enabled more robust normal vector calculation. In comparison, inclination angles derived from multi-view reconstructed point clouds (Fig. 10e) achieved an $R^{2}$ of 0.9912, which is slightly higher than our completion method but requires denser, multi-view input data that is not always available in field phenotyping scenarios.

## 4 Discussion

In this study, we propose a non-rigid transformation-based data augmentation method to enrich the morphological diversity of plant point clouds. The proposed MSDDG framework provides a general paradigm and structural solution for plant point cloud completion. The core of this paradigm lies in “multi-scale geometric feature extraction + dual-discriminator adversarial supervision”, which is not bound to a specific plant species or growth stage, but focuses on capturing the inherent structural and morphological distribution patterns of plants. This general design ensures that the framework has strong adaptability and scalability for different agricultural phenotyping scenarios. Based on this approach, we construct a novel dataset for plant point cloud completion, named Plant4L. During the data preprocessing stage, we employ virtual cameras to simulate different viewpoints and to generate corresponding projected silhouette images of the incomplete point clouds. These silhouettes serve as auxiliary inputs for network training, rather than being part of the dataset itself.

Furthermore, we introduce MSDDG (Multi-scale Dual-Discriminator GAN), a generative adversarial network designed for plant point cloud completion. MSDDG comprises a multi-scale point cloud generator (MSPG) and two discriminators: a multi-view projected silhouette discriminator and a spatial distance discriminator. These dual discriminators guide the adversarial training to enhance the generator's ability to recover complete plant structures from partial inputs. This design enables the generator to produce completions that are both geometrically accurate and visually consistent from multiple perspectives.

During the training of the MSDDG network, the loss function did not exhibit any obvious signs of non-convergence, indicating that the model possesses a strong ability to learn the geometric features and structural patterns of point clouds. Analysis of the completion results on the validation set shows that the proposed model can accurately recover the missing parts of plant point clouds, with particularly impressive performance in structurally distinctive regions such as stems and leaves. In Fig. 7, Fig. S6, Fig. S7 and Fig. S8, the pumpkin exhibits a relatively simple spatial structure with the fewest number of leaves among the plant species shown, whereas the luffa demonstrates the most complex morphology with the highest leaf density. As observed from the completion results of the four plant categories in VMV-1 and VMV-2, when the missing regions are spatially continuous and concentrated, the completed point clouds exhibit a well-distributed reconstruction across both local and global structures. This can be attributed to the multi-resolution encoder (MRE), which maintains feature consistency by cascading local and global features during the encoding process. Furthermore, the completed shapes accurately preserve the contour and morphological characteristics of the original plants, benefiting from the design of the dual-discriminator, which enforces consistency both in spatial point distributions and across multi-view projected silhouettes. This enables the generator to produce point clouds that closely align with the original plant structure. However, in VMV-3, VMV-4, and VMV-5, the incomplete point clouds exhibit non-continuous and spatially scattered missing regions due to optical occlusions. For instance, the luffa in VMV-3, the eggplant and luffa in VMV-4, and the pumpkin and sunflower in VMV-5 all suffer from fragmented occlusions that are not topologically coherent with the plant's structural distribution. Although the completion results in these cases still reflect an overall plausible 3D reconstruction, the generator (MSPG) tends to concentrate the predicted points in one dominant region, leaving other missing areas with significantly fewer completions. This limitation arises from the fixed number of output points defined by the MSPG architecture. When the missing regions are dispersed across multiple areas, a higher number of points is generally required to adequately represent the incomplete regions. However, a generator constrained to produce a fixed number of points struggles to distribute them effectively across multiple missing zones. These observations highlight both the strengths and limitations of the proposed MSDDG framework: it performs well under conditions of concentrated and continuous occlusions, but exhibits reduced flexibility and coverage when faced with widely scattered missing areas due to the fixed-size point cloud generation constraint.

The experimental results presented in Table 1 and Fig. S 9 reveal significant differences in point cloud completion performance among the evaluated methods. These differences can be largely attributed to the architectural characteristics and design assumptions inherent in each network. PF-Net, which relies heavily on global feature encoding to guide completion, struggles in scenarios with sparse or dispersed missing regions. Its lack of effective multi-scale feature fusion leads to poor local detail recovery and causes the predicted points to deviate from actual plant morphology—especially in geometrically complex structures like Eggplant and Luffa. Additionally, PF-Net tends to generate completions that extend beyond the natural plant boundary due to insufficient spatial constraint in its reconstruction process. FoldingNet, by contrast, employs a fixed 2D grid folding mechanism to reconstruct 3D structures. While this approach allows for regular and continuous outputs in simpler shapes such as Pumpkin, its structural rigidity limits adaptability to diverse plant geometries. The network often produces point distributions that are either overly concentrated or dispersed away from the true missing regions, especially when the input contains irregular occlusions. SA-Net integrates attention mechanisms to aggregate local and global features, which helps in preserving coarse structural alignment. However, its attention modules often fail to maintain consistent spatial reasoning across complex plant topologies, resulting in incomplete or spatially misaligned reconstructions. This is particularly evident in the Sunflower case, where the predicted shapes approximate the ground truth but are offset from the true missing regions.

In contrast, our proposed MSDDG framework demonstrates superior performance across all plant categories and occlusion types, which can be attributed to several key architectural innovations. First, the integration of a multi-resolution encoder enables the effective extraction and fusion of both global context and local geometric details, which is essential for handling the varying spatial scales present in plant structures. Second, the multi-scale point cloud generator allows for adaptive and fine-grained point generation, reducing the risk of structural overshooting or underfitting. Most critically, the dual-discriminator strategy—which evaluates the consistency of generated point clouds from both spatial and multi-view silhouette perspectives—serves as a strong supervisory signal, enforcing adherence to realistic plant morphology during training. These design choices collectively address the limitations observed in prior methods and enable MSDDG to perform robustly in both spatially continuous and dispersed missing region scenarios. The network not only enhances reconstruction completeness but also ensures structural fidelity and spatial alignment, making it well-suited for real-world plant phenotyping applications where shape diversity and partial observations are common.

The single-view 3D reconstruction results using the pretrained MSPG module reveal promising capabilities in point cloud completion for previously unseen plant categories. The network demonstrates the ability to recover major structural elements from partial observations, particularly in cases where missing regions are spatially concentrated and the overall morphology is relatively simple. This suggests that the model has learned robust shape priors from the Plant4L dataset, which can generalize to a certain extent across different plant types. The observed improvements in 3D completeness and structural plausibility can be attributed to the multi-scale feature representation learned during pretraining. These features enable the model to infer plausible geometric patterns even from limited input, maintaining overall contour consistency. The network's successful reconstructions in views (1) and (4) support its capacity for effective shape completion under moderate occlusion.

However, several limitations emerge when the model is applied to highly incomplete inputs or plant categories not seen during pretraining. First, when the missing regions are extensive or widely dispersed—as observed in views (2) and (3)—the network tends to produce clustered completions that fail to uniformly cover all missing areas. This limitation likely stems from the fixed number of output points and the lack of explicit spatial priors guiding completion across disconnected regions. Second, when transferred to novel plant types, MSPG shows a decline in accuracy and geometric alignment, indicating a drop in cross-category generalization. This is likely due to distributional differences in plant structures not represented in the training data, suggesting a form of overfitting to the Plant4L morphology space.

These findings highlight both the potential and the boundaries of pretrained completion models in cross-domain settings. Future improvements may include incorporating adaptive output mechanisms, integrating semantic priors to guide completion across structurally diverse categories, or leveraging multi-view consistency constraints even in single-view inference scenarios.

Although the proposed completion method achieves promising results in many aspects, certain limitations remain. For instance, the completion performance is still unstable in extremely sparse regions or areas with severe structural occlusion. Furthermore, the model training relies heavily on well-annotated paired point clouds, which restricts its applicability in large-scale unsupervised scenarios.

The network has learned inherent contour rules and geometric spatial distribution priors of plants, rather than memorizing growth-stage-specific features of juvenile samples. The MRE extracts view-invariant and growth-stage-invariant geometric features, such as leaf curvature, stem-leaf connection topology, and organ spatial distribution patterns, which are common across different growth stages of plants with similar morphological types. This enables the model to maintain reliable completion performance for plants with similar stem-leaf structures, even for adult plants with more complex canopy structures, as long as the morphological priors match the learned features.

The limitations of our network architecture are primarily related to the size and morphological diversity of the training dataset. When faced with plants exhibiting highly complex structures, the completion results may be less satisfactory. Additionally, due to constraints on the number of points that can be predicted, the model struggles to generate predictions simultaneously across multiple missing regions when the occlusions are extensive. As a result, the predicted completions tend to exhibit spatial continuity, which may not fully capture the true distribution of missing parts in such challenging cases.

## 5 Conclusion

This paper proposes a novel plant point cloud completion method based on a generative adversarial network (MSDDG), along with a new dataset (Plant4L), to address the common issue of missing data in plant 3D reconstruction caused by optical occlusions. By constructing the Plant4L dataset using non-rigid transformations and a point cloud completion strategy, the study enables more accurate modeling of occluded plant structures. A Multi-Resolution Encoder (MRE) was employed to extract both global and local geometric features of plants. The proposed MSDDG model incorporates a Multi-Scale Point cloud Generator (MSPG), a spatial distance discriminator, and a multi-view projection silhouette discriminator. Extensive experiments on both the validation set and application for 3D reconstruction from single view demonstrate that MSDDG significantly improves the completeness of reconstructed 3D plant models.

While MSDDG achieves promising results in plant point cloud completion, its high model complexity requires extensive data and pretraining. Future work may explore lightweight architectures for instance segmentation and broader data augmentation to improve efficiency and generalization. This study provides methodological support for plant phenotyping, digital modeling, and ecological monitoring, offering both theoretical and practical value. Further research may investigate unsupervised completion and incorporate structural priors based on geometric relationships among plant components (e.g., stems and leaves).

## Author contributions

**Qingguang Chen:** Writing – review & editing, Writing – original draft, Supervision, Methodology, Funding acquisition, Conceptualization. **Jiajin Liu:** Writing – review & editing, Writing – original draft, Methodology, Formal analysis, Conceptualization. **Hongwei Sun**: Methodology, Formal analysis, Conceptualization. **Ting Sun**: Methodology, Formal analysis, Conceptualization. **Wenyu Zhang**: Methodology, Formal analysis, Conceptualization. **Hang Lu:** Software, Investigation. **Yingying Pan:** Software, Investigation. **Wenhan Luo:** Investigation. **Lianjie Chen:** Resources, Methodology, Investigation. **Jie Ying:** Resources, Methodology, Investigation. **Simin Kang:** Resources, Methodology, Investigation. **Jingcheng Zhang:** Resources, Methodology, Investigation. **Kaihua Wu:** Resources, Methodology, Investigation.

## Funding

This research was supported by 10.13039/501100012166National Key R&D Program of China (2022YFD2000100), Pioneer and Leading Goose R&D Program of Zhejiang (2022C02026), Zhejiang Provincial Natural Science Foundation of China under Grant No. (LTGY23F030001).

## Declaration of competing interest

The authors declare that they have no known competing financial interests or personal relationships that could have appeared to influence the work reported in this paper.

## Figures and Tables

**figs1:**
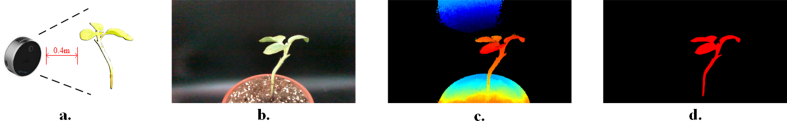


**figs2:**
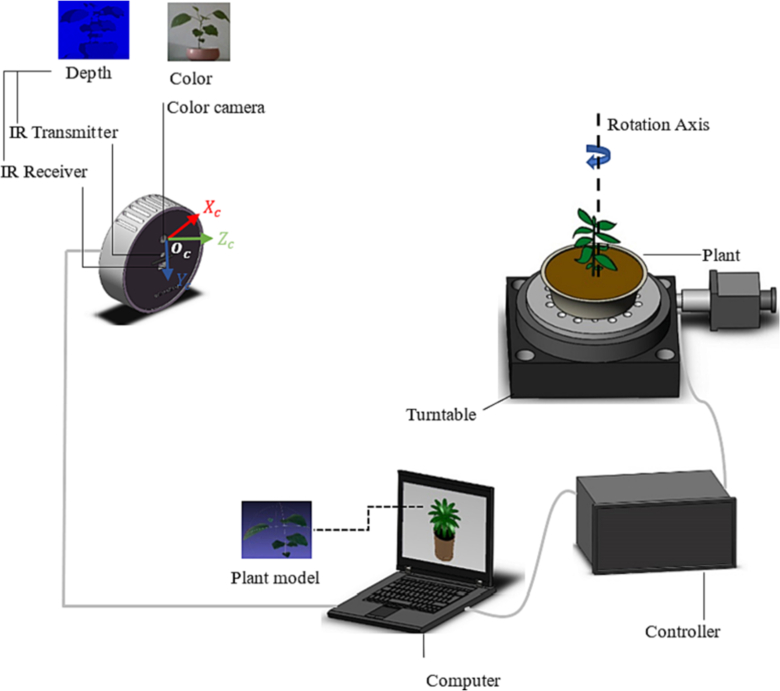


**figs3:**
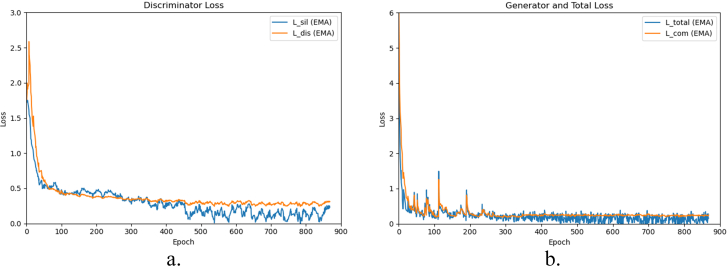


**figs4:**
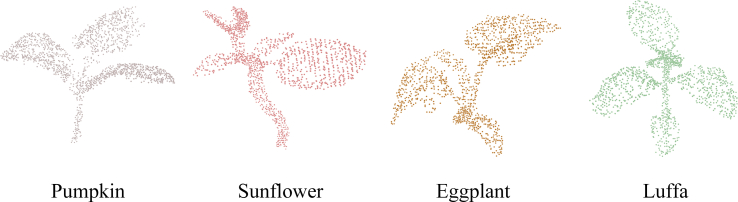


**figs5:**
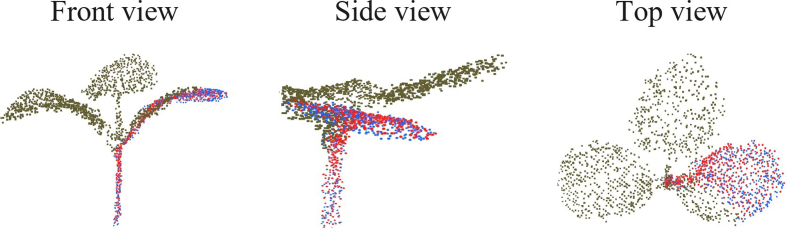


**figs6:**
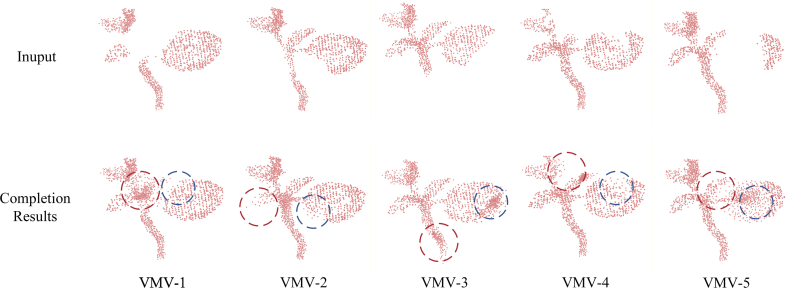


**figs7:**
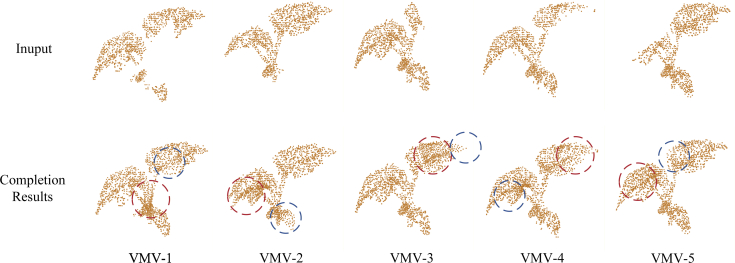


**figs8:**
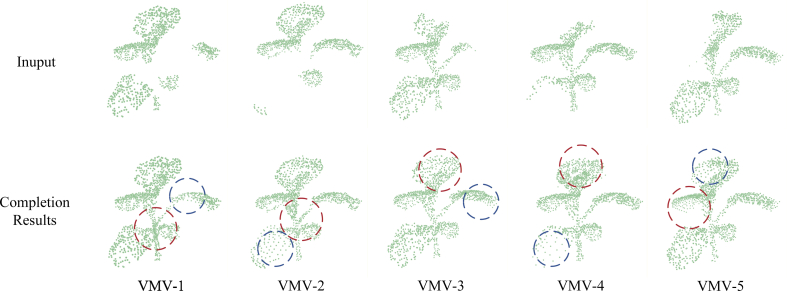


**figs9:**
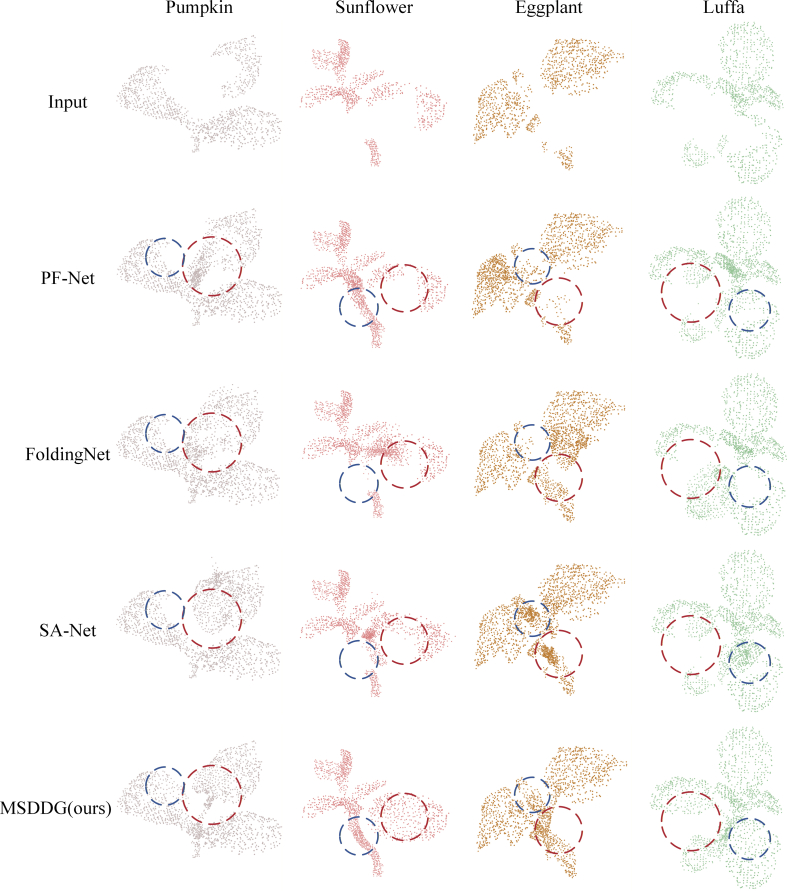


**figs10:**
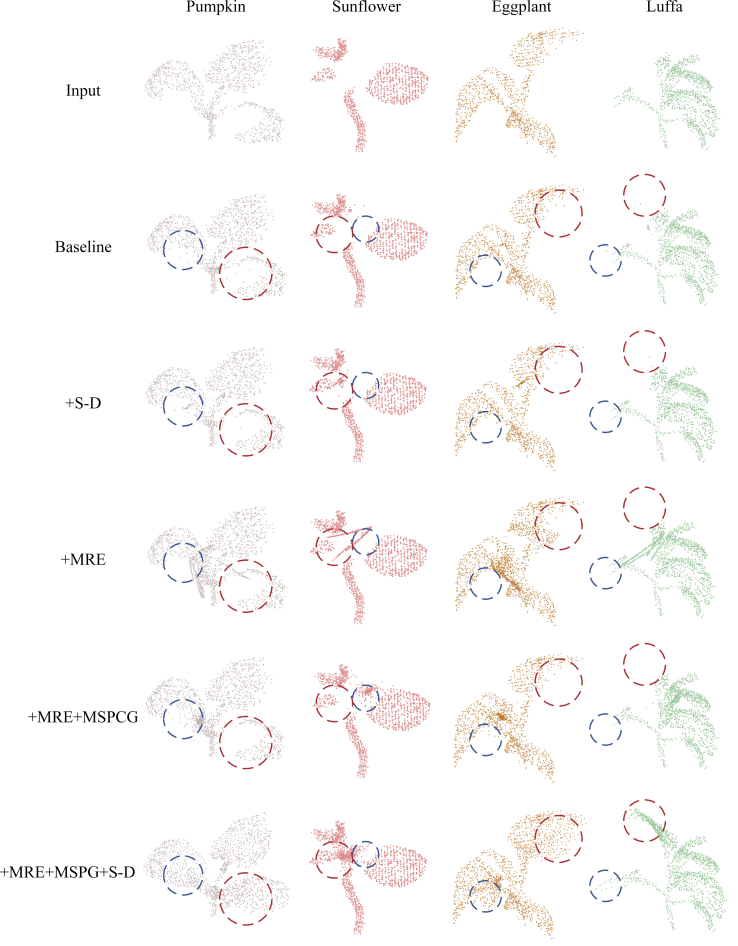


**figs11:**
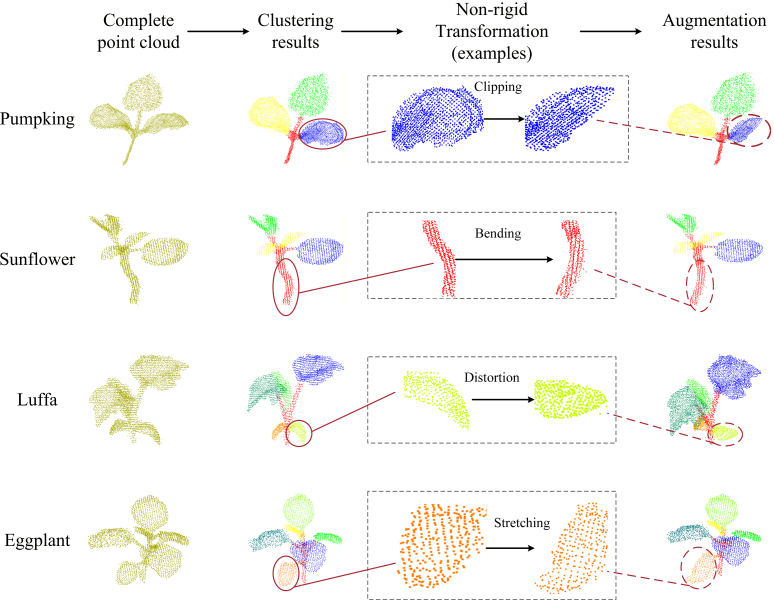


**figs12:**
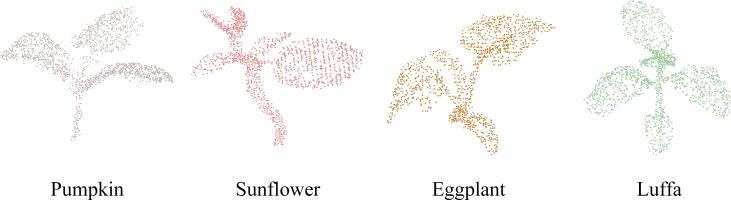


**figs13:**
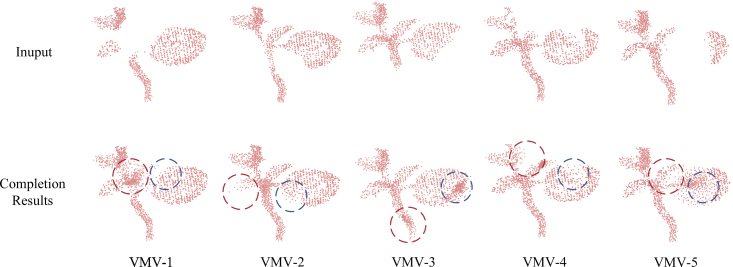


**figs14:**
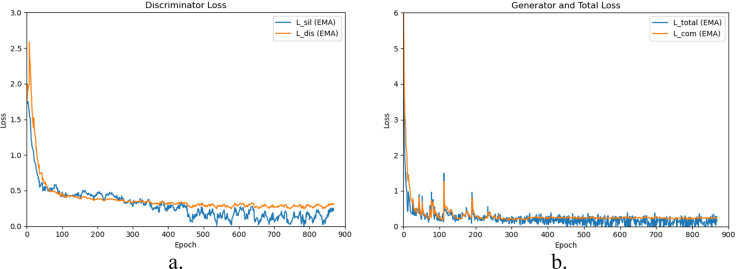


**figs15:**
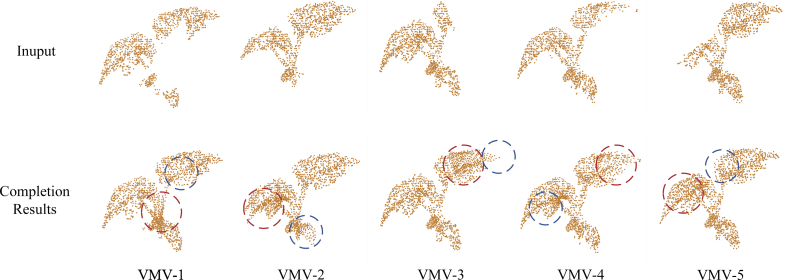


**figs16:**
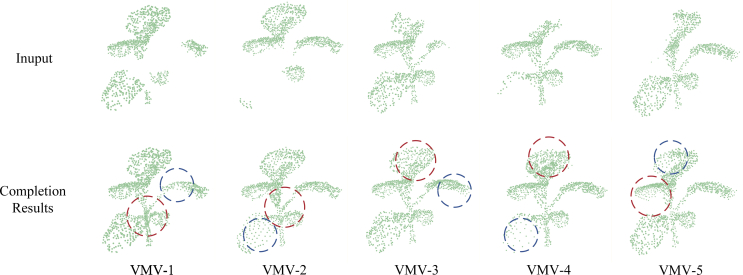


**figs17:**
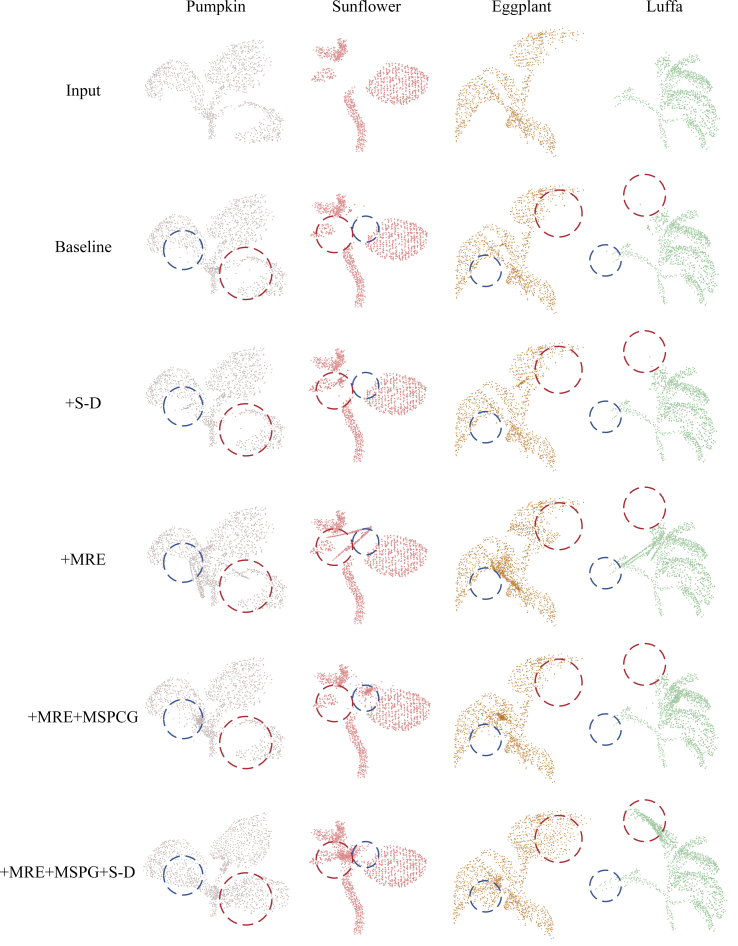


**figs18:**
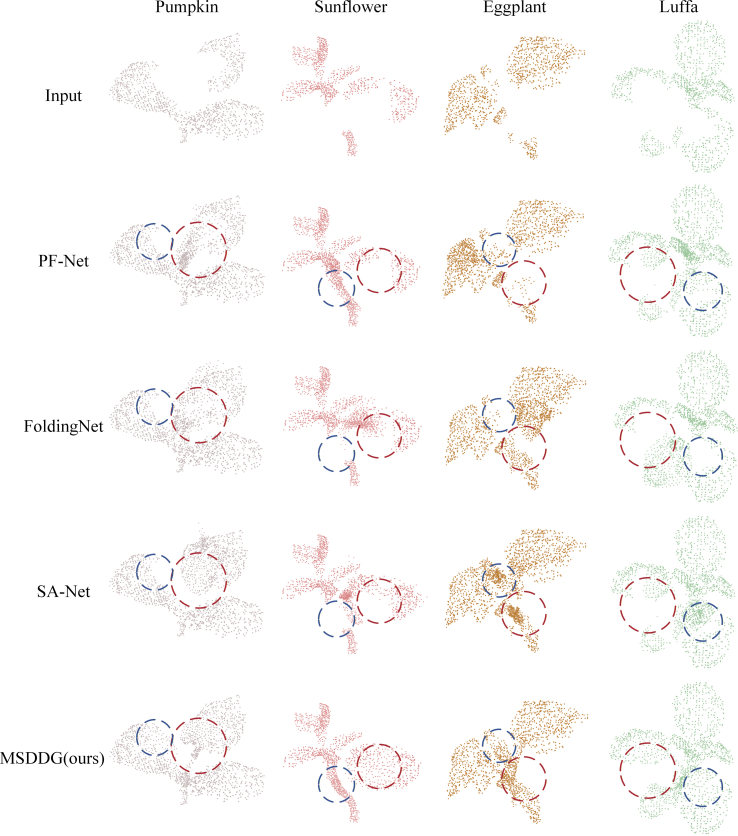


**figs19:**
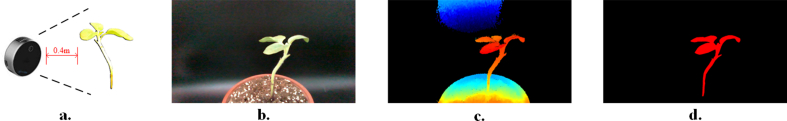


**figs20:**
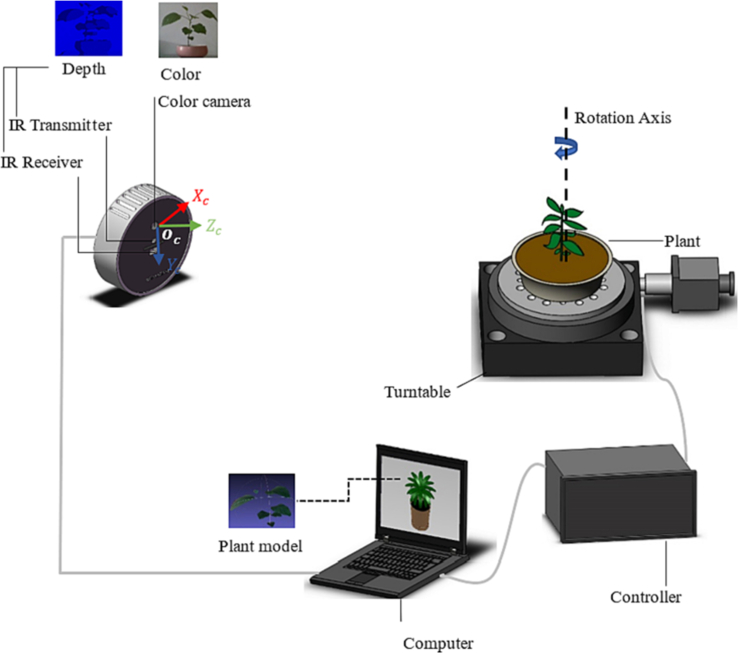


## Data Availability

The source code and datasets used in this study are publicly available at https://github.com/Amuro-Aznable/MSCGPCN.git.

## References

[1] Tardieu F., Cabrera-Bosquet L., Pridmore T., Bennett M. (2017). Plant phenomics, from sensors to knowledge. Curr. Biol..

[2] Hui F., Zhu J., Hu P., Meng L., Zhu B., Guo Y., Li B., Ma Y. (2018). Image-based dynamic quantification and high-accuracy 3D evaluation of canopy structure of plant populations. Ann. Bot..

[3] Li Y., Liu J., Zhang B., Wang Y., Yao J., Zhang X., Fan B., Li X., Hai Y., Fan X. (2022). Three-dimensional reconstruction and phenotype measurement of maize seedlings based on multi-view image sequences. Front. Plant Sci..

[4] Wu S., Wen W., Wang Y., Fan J., Wang C., Gou W., Guo X. (2020). MVS-pheno: a portable and low-cost phenotyping platform for maize shoots using multiview stereo 3D reconstruction. Plant Phenomics.

[5] Zhu T., Ma X., Guan H., Wu X., Wang F., Yang C., Jiang Q. (2023). A calculation method of phenotypic traits based on three-dimensional reconstruction of tomato canopy. Comput. Electron. Agric..

[6] Chaudhury A., Ward C., Talasaz A., Ivanov A.G., Brophy M., Grodzinski B., Hüner N.P., Patel R.V., Barron J.L. (2018). Machine vision system for 3D plant phenotyping. IEEE ACM Trans. Comput. Biol. Bioinf.

[7] Milella A., Marani R., Petitti A., Reina G. (2019). In-field high throughput grapevine phenotyping with a consumer-grade depth camera. Comput. Electron. Agric..

[8] Zheng Y., Qi H., Li L., Li S., Huang Y., He C., Wang D. (2024). Motion-guided and occlusion-aware multi-object tracking with hierarchical matching. Pattern Recogn..

[9] Laga H., Jospin L.V., Boussaid F., Bennamoun M. (2020). A survey on deep learning techniques for stereo-based depth estimation. IEEE Trans. Pattern Anal. Mach. Intell..

[10] Henry P., Krainin M., Herbst E., Ren X., Fox D. (2012). RGB-D mapping: using kinect-style depth cameras for dense 3D modeling of indoor environments. Int. J. Robot Res..

[11] Wang X., Ang M.H., Lee G.H. (2021). Proceedings of the IEEE/CVF International Conference on Computer Vision.

[12] Curless B., Levoy M. (1996). Proceedings of SIGGRAPH.

[13] Das Choudhury S., Maturu S., Samal A., Stoerger V., Awada T. (2020). Leveraging image analysis to compute 3D plant phenotypes based on voxel-grid plant reconstruction. Front. Plant Sci..

[14] Yang L., Zhai R., Yang X. (2019). Segmentation of plant organs point clouds through super voxel-based region growing methodology. Comput. Eng. Applicat..

[15] Qi C.R., Su H., Mo K., Guibas L.J. (2017). Proceedings of the IEEE Conference on Computer Vision and Pattern Recognition.

[16] Qi C.R., Yi L., Su H., Guibas L.J. (2017). PointNet++: deep hierarchical feature learning on point sets in a metric space. Adv. Neural Inf. Process. Syst..

[17] Yuan W., Khot T., Held D., Mertz C., Hebert M.P.C.N. (2018). Proceedings of 3DV.

[18] Pan L., Chen X., Cai Z., Zhang J., Zhao H., Yi S., Liu Z. (2021). Proceedings of the IEEE/CVF Conference on Computer Vision and Pattern Recognition.

[19] Zhang J., Chen X., Cai Z., Pan L., Zhao H., Yi S., Yeo C.K., Dai B., Loy C.C. (2021). Proceedings of the IEEE/CVF Conference on Computer Vision and Pattern Recognition.

[20] Sarmad M., Lee H.J., Kim Y.M. (2019). Proceedings of the IEEE/CVF Conference on Computer Vision and Pattern Recognition.

[21] Yang Y., Feng C., Shen Y., Tian D. (2018). Proceedings of the IEEE Conference on Computer Vision and Pattern Recognition.

[22] Wen X., Li T., Han Z., Liu Y.S. (2020). Proceedings of the IEEE/CVF Conference on Computer Vision and Pattern Recognition.

[23] Huang Z., Yu Y., Xu J., Ni F., Le X. (2020). Proceedings of the IEEE/CVF Conference on Computer Vision and Pattern Recognition.

[24] Li K., Zhao W., Liu J., Wang J., Zhang H., Jiang H. (2024). FuNet: multi-feature fusion for point cloud completion network. Electronics.

[25] Li X., Zhou Z., Xu Z., Jiang H., Zhao H. (2020). Proceedings of the Sixth Symposium on Novel Optoelectronic Detection Technology and Applications.

[26] Zeng A., Jiewei P., Chang L., Dan P., Yanrong J., Xiaobo Z. (2022). Plant point cloud completion network based on multi-scale geometry-aware point transformer. Trans. Chin. Soc. Agric. Eng..

[27] Chen H., Liu S., Wang C., Wang C., Gong K., Li Y., Lan Y. (2023). Point cloud completion of plant leaves under occlusion conditions based on deep learning. Plant Phenomics.

[28] Guo Z., Tang Y., Zhang R., Wang D., Wang Z., Zhao B., Li X. (2023). Proceedings of the IEEE/CVF International Conference on Computer Vision.

[29] Yang J., Deng H., Zhang Y., Zhou Y., Miao T. (2024). Application of amodal segmentation for shape reconstruction and occlusion recovery in occluded tomatoes. Front. Plant Sci..

[30] Lou M., Lu J., Wang L., Jiang H., Zhou M. (2022). Growth parameter acquisition and geometric point cloud completion of lettuce. Front. Plant Sci..

[31] Li Y., Si S., Liu X., Zou L., Wu W., Liu X., Zhang L. (2023). Three-dimensional reconstruction of cotton plant with internal canopy occluded structure recovery. Comput. Electron. Agric..

[32] Wei K., Liu S., Chen Q., Huang S., Zhong M., Zhang J., Sun H., Wu K., Fan S., Ye Z. (2024). Fast multi-view 3D reconstruction of seedlings based on automatic viewpoint planning. Comput. Electron. Agric..

[33] Chen Q., Huang S., Liu S., Zhong M., Zhang G., Song L., Kong D. (2024). Multi-view 3D reconstruction of seedling using 2D image contour. Biosyst. Eng..

[34] Liu X., Liu X., Li Y., Yuan J., Song L., Li H., Wu M. (2020). Estimation model of canopy stratification porosity based on morphological characteristics: a case study of cotton. Biosyst. Eng..

[35] Martínez-Force E., Dunford N.T., Salas J.J. (2015).

[36] Zolix Instrument Co (2016). https://www.zolix.com.cn/prodcon_371_384_447_894.html.

[37] Sorkine O., Alexa M. (2007). Symposium on Geometry Processing.

[38] Baek I., Kanda A., Tai T.C., Saxena A., Rajkumar R. (2019). Proceedings of the IEEE Intelligent Vehicles Symposium.

[39] Wu L., Cheng X., Hou J., Xu Y., Zeng H. (2024). Self-supervised 3D point cloud completion via multi-view adversarial learning. arXiv.

[40] Loshchilov I., Hutter F. (2017). Decoupled weight decay regularization. arXiv.

[41] Ronneberger O., Fischer P., Brox T. (2015). MICCAI.

[42] Zhou Z., Siddiquee M.M.R., Tajbakhsh N., Liang J. (2018). Deep Learning in Medical Image Analysis and Multimodal Learning for Clinical Decision Support.

[43] Chang A.X., Funkhouser T., Guibas L., Hanrahan P., Huang Q., Li Z., Yu F. (2015). ShapeNet: an information-rich 3D model repository. arXiv.

[44] Kimara E., Hadadi M., Godbersen J., Balu A., Jubery T.Z., Li Y., Krishnamurthy A., Schnable P.S., Ganapathysubramanian B. (2025 Dec). MaizeField3D: a curated 3D point cloud and procedural model dataset of field-grown maize from a diversity panel. Plant Phenomics.

