# Supplementary material for: MSDDG: Multi-scale dual-discriminator GAN for point cloud completion of plant
Source: Plant Phenomics. 2026 Apr 23;8(2):100218. doi: 10.1016/j.plaphe.2026.100218 (PMC13157219; doi:10.1016/j.plaphe.2026.100218)
Supplement: Multimedia component 1 [file mmc1.docx]

# Data availability

The source code and datasets used in this study are publicly available at <https://github.com/Amuro-Aznable/MSCGPCN.git>.

# Supplementary Materials

**Fig S1.** Initial 3D point cloud data acquisition of the plant. **a**. perspective of data acquisition, **b**. RGB image of plant, **c**. depth map represented by color where redder color indicates closer proximity to the sensor, **d**. mask segmented from the RGB image.

**Fig S2.** Illustration of our 3D reconstruction system during data acquisition.

**Fig S3**. The decreasing trend of multi-objective loss functions during training. **a**. Discriminator loss showing $L_{dis}$ and $L_{sil}$ over epochs. **b**. Generator and total loss showing $L_{com}$ and Total ($L$), illustrating convergence behavior.

**Fig S4**. The representative plant point clouds for each category in the Plant4L dataset.

**Fig S5.** Point cloud completion results of a sample plant observed from three viewpoints.

**Fig S6.** The visualization of the plant point cloud completion for Plant4L(Sunflower).

**Fig S7.** The visualization of the plant point cloud completion for Plant4L(Eggplant).

**Fig S8.** The visualization of the plant point cloud completion for Plant4L(Luffa).

**Fig S 9** Comparison methods of the plant point cloud for the completed visualization under same view. The brown points represent the incomplete point clouds, blue points represent the ground truth of missing region, and red points represent the value of missing region predicted by MSDDG.

**Fig S10.** Ablation visualization results of the proposed module.

**Fig S11.** The process of constructing the dataset only shows a portion of the data for each category. For each plant point cloud, from different clustering results (where different colors correspond to different categories), different shapes of non-rigid transformation results were obtained. The part circled in red in the figure is an enlarged display of the comparison before and after the non-rigid transformation.

**Table S1** Quantitative evaluation metrics of MSDDG completion performance under different rotation conditions (Pumpkin validation set)

| **Rotation Condition** | $\boldsymbol{d}_{\boldsymbol{CD}}$ | $\boldsymbol{d}_{\boldsymbol{UCD}}$ | $\boldsymbol{F}_{\boldsymbol{1}}$ |
| --- | --- | --- | --- |
| \| Baseline (No rotation) \| \| --- \| \| X-axis (30°/60°/90°) \| \| Y-axis (30°/60°/90°) \| \| Z-axis (30°/60°/90°) \| \| Max fluctuation \| | \| 0.211 \| \| --- \| \| 0.211±0.000 \| \| 0.213±0.000 \| \| 0.211±0.000 \| \| +0.002 \| | \| 0.155 \| \| --- \| \| 0.155±0.000 \| \| 0.155±0.000 \| \| 0.155±0.000 \| \| +0.001 \| | \| 0.903 \| \| --- \| \| 0.903±0.000 \| \| 0.903±0.002 \| \| 0.902±0.000 \| \| -0.002 \| |

**Table S2** Ablation experiments of number of multi-view silhouette projection in multi-view projected silhouette discriminator.

| Number | $d_{CD}$ | $d_{UCD}$ | $d_{HD}$ | $F_{1}$ |
| --- | --- | --- | --- | --- |
| 4 | 0.793 | 0.446 | 0.954 | 0.036 |
| 6 | 0.623 | 0.395 | 0.781 | 0.282 |
| 8 | 0.529 | 0.368 | 0.513 | 0.424 |
| **10** | **0.187** | **0.309** | **0.462** | **0.906** |
| 12 | 0.408 | 0.331 | 0.674 | 0.774 |
