# Supplementary figures and images for: MSDDG: Multi-scale dual-discriminator GAN for point cloud completion of plant
Source: Plant Phenomics. 2026 Apr 23;8(2):100218. doi: 10.1016/j.plaphe.2026.100218 (PMC13157219; doi:10.1016/j.plaphe.2026.100218)

Front view

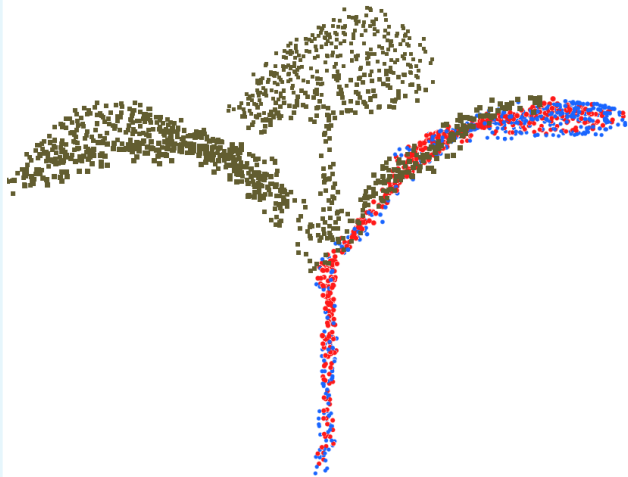

Side view

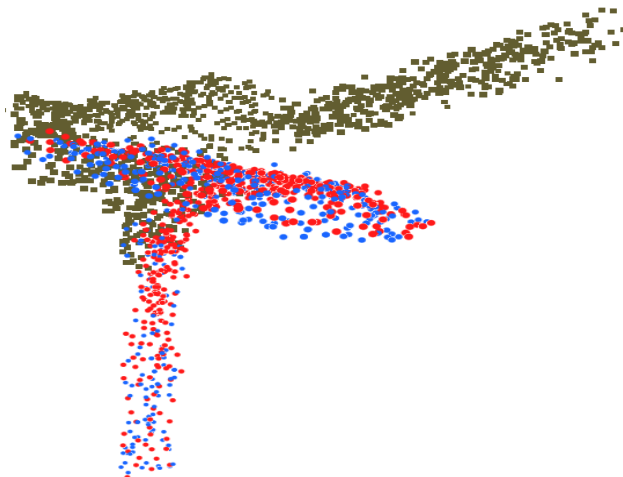

Top view

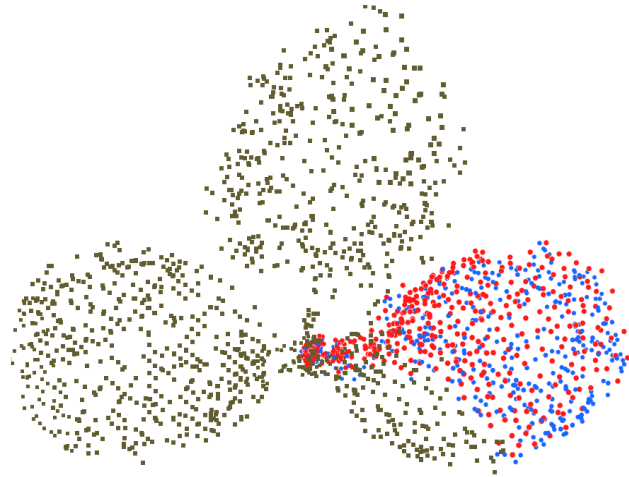

Supplement: Multimedia component 2 [file mmc2.pdf]
