# Supplementary material for: MSDDG: Multi-scale dual-discriminator GAN for point cloud completion of plant
Source: Plant Phenomics. 2026 Apr 23;8(2):100218. doi: 10.1016/j.plaphe.2026.100218 (PMC13157219; doi:10.1016/j.plaphe.2026.100218)
Supplement: Multimedia component 3 [file mmc3.pdf]

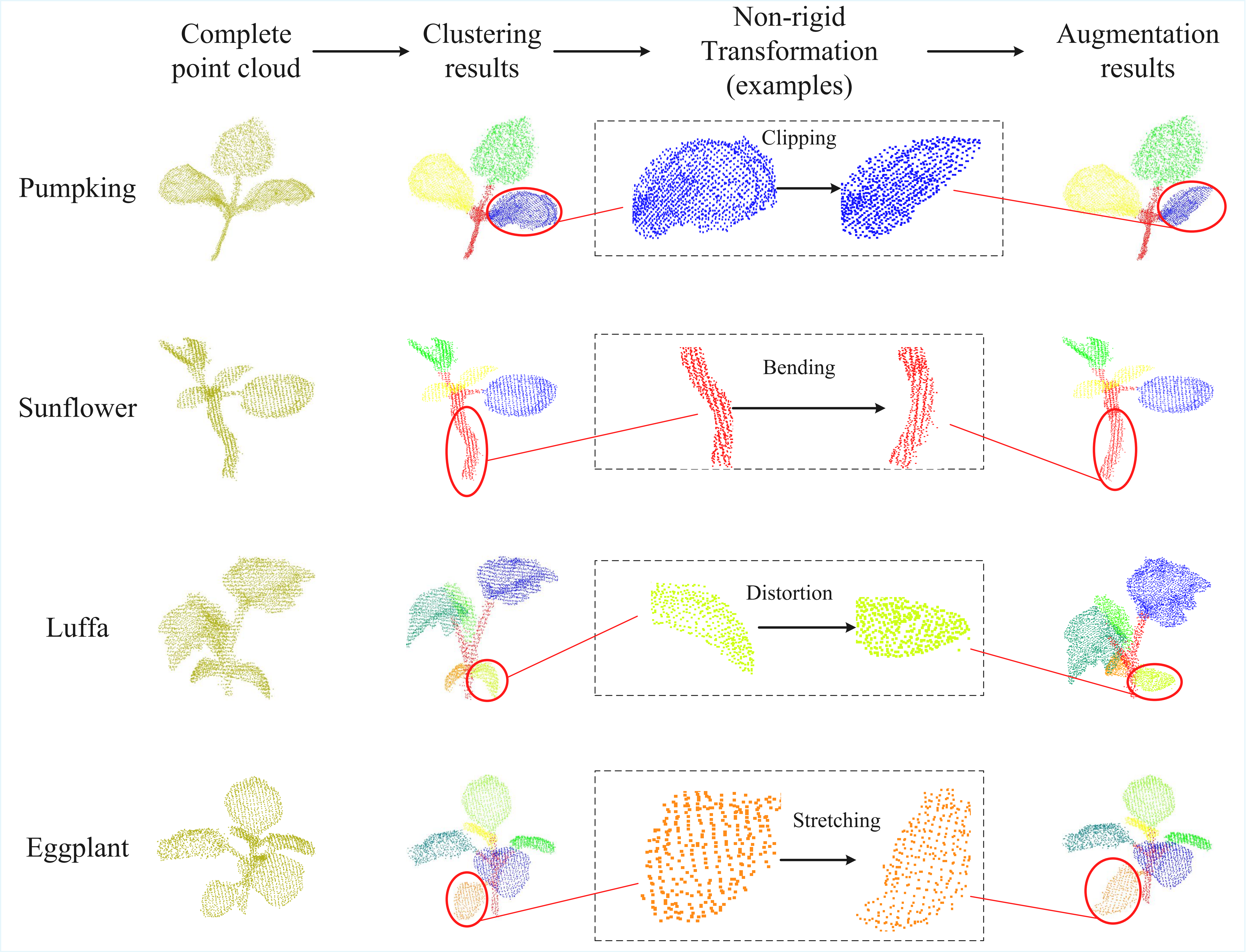
Complete point cloud
Clustering results
Non-rigid Transformation (examples)
Augmentation results
Pumpking
Clipping
Sunflower
Bending
Luffa
Distortion
Eggplant
Stretching
